# Engineering the Crystalline Architecture for Enhanced Properties in Fast-Rate Processing of Poly(ether ether ketone) (PEEK) Nanocomposites

**DOI:** 10.1021/acsaenm.4c00217

**Published:** 2024-08-07

**Authors:** Behrooz Shirani Bidabadi, Emile Motta de Castro, Mia Carrola, Pratik Koirala, Mehran Tehrani, Amir Asadi

**Affiliations:** ‡ Department of Engineering Technology and Industrial Distribution, Texas A&M University, College Station, Texas 77843-3367, United States; § J. Mike Walker ’66 Department of Mechanical Engineering, Texas A&M University, College Station, Texas 77843, United States; ∥ Department of Materials Science and Engineering, Texas A&M University, College Station, Texas 77843, United States; ⊥ Walker Department of Mechanical Engineering, University of Texas at Austin, Austin, Texas 78712-1591, United States; ¶ Department of Structural Engineering, University of California at San Diego, La Jolla, California 92093, United States; # Program in Materials Science and Engineering, University of California at San Diego, La Jolla, California 92093,United States

**Keywords:** crystallization kinetics, crystalline morphology and architecture, poly(aryl ether ether ketone), nanocomposite, cellulose nanocrystals, graphene nanoplatelets

## Abstract

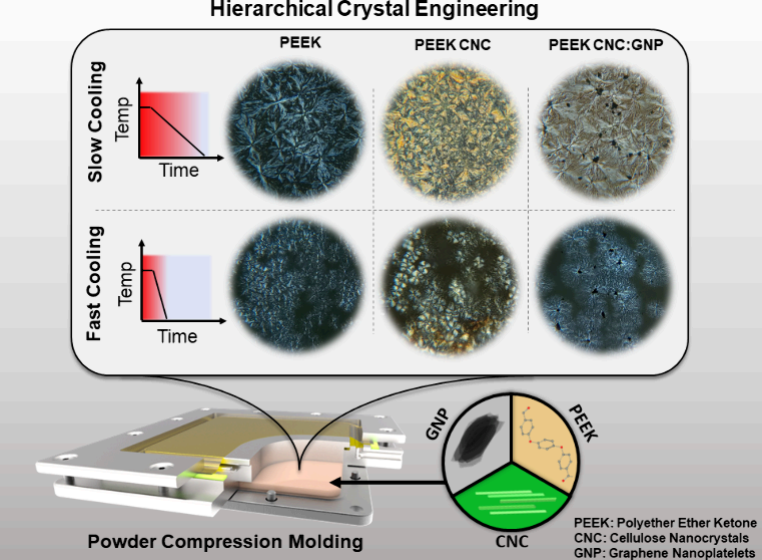

Rapid cooling in fast-rate manufacturing processes such as additive manufacturing and stamp forming limits the development of crystallinity in semicrystalline polymer nanocomposites and, therefore, potential improvements in the mechanical performance. While the nucleation, chain mobility, and crystallization time from rapid cooling are known competing mechanisms in crystallization, herein we elucidate that the crystalline morphology and architecture also play a key role in tuning the mechanical performance. We explore how modifying the spherulite morphology via a cellulose nanocrystal (CNC) and graphene nanoplatelet (GNP) hybrid system in their pristine form can improve or preserve the mechanical properties of poly(ether ether ketone) (PEEK) nanocomposites under two extreme cooling rates (fast −460 °C/min and slow −0.7 °C/min). A scalable manufacturing methodology using water as the medium to disperse the powder system was developed, employing a CNC as a dispersing agent and stabilizer for PEEK and GNP. Despite the expected limited mechanical reinforcement due to thermal degradation, CNCs significantly impacted PEEK’s crystalline architecture and mechanical performance, suggesting that surface interactions via lattice matching with PEEK’s (200) crystallographic plane play a critical role in engineering the microstructure. In fast cooling, the CNC and CNC:GNP systems reduced the crystallinity, respectively, yet led to minimizing the reduction in the tensile strength and maintaining the tensile modulus at the Neat level in slow cooling. With slow cooling, crystallinity remained relatively unchanged; however, the addition of CNC:GNP improved the strength and modulus by ∼10% and ∼16%, respectively. These findings demonstrate that a hybrid nanomaterial system can tailor PEEK’s crystalline microstructure, thus presenting a promising approach for enhancing the mechanical properties of PEEK nanocomposites in fast-rate processes.

## 1 Introduction

Developing sustainable, weldable, chemical- and temperature-resistant composites with high mechanical and functional (thermal/electrical) properties is critical to the next generation of structural materials for aerospace applications.^1−3^ Poly(ether ether ketone) (PEEK), an aromatic high-performance thermoplastic, can achieve these goals in combination with various fillers—either through carbon fibers or nanoparticles like carbon nanotubes (CNTs) and graphene—to achieve the missing mechanical or functional properties.^4^ Yet, manufacturing PEEK composites requires overcoming set challenges given PEEK’s inherent mechanical rigidity and chemical and thermal stability attributed to its aromatic backbone. As a semicrystalline polymer, it is well-known that the cooling rate from melt and annealing of PEEK affect its crystallinity and therefore mechanical properties,^5,6^ which can, in turn, serve as an excellent feature for tailoring mechanical response for a specific application. This suggests, however, that PEEK’s mechanical properties and dimensional accuracy are sensitive to its thermal history. While slower cooling rates from melt improve the crystallinity, they prolong the fabrication times and limit production rates. Controlling the PEEK crystallinity is especially critical in injection molding,^7^ additive manufacturing,^8^ and automated fiber placement,^9^ where nonuniform crystallization occurs due to high cooling rates induced from low mold/ambient temperatures. If tuning the crystallinity is a significant factor in the final mechanical properties, how can we engineer the microstructure without inadvertently modifying the chemical properties to tailor the desired final performance of PEEK? Aside from the cooling rate, the controlled introduction of nanomaterials provides a secondary mechanism by which the crystalline structure can be tuned, which can serve to improve the consistency and performance of rapidly manufactured PEEK components.

Many studies have previously shown that the reinforcement mechanism behind the addition of nanomaterials can be complex and often cannot be explained by simple rule-of-mixtures-type analysis alone.^10−12^ While homogeneous dispersion is the most well-known issue that limits the effectiveness of fillers for both the mechanical and functional properties, the fillers themselves also play a role in dictating the crystalline microstructure because they can introduce ordered transcrystalline regions, improve the crystallization rate by the addition of nucleation points, or hinder crystallization by limiting the chain mobility. Because the mechanical properties of thermoplastics are dictated by chain mobility and entanglement, it becomes critical to control the microstructural changes imparted by nanomaterials. Extensive work throughout literature has been done to provide a generalized understanding of the effect of different types of fillers—ceramics^13,14^ graphene,^14,15^ graphene nanoplatelets (GNPs),^4,16^ and CNTs^12,17−20^—on the multifunctional properties and crystallization kinetics of semicrystalline thermoplastics. These studies generally find that while the rate of crystallization and mechanical properties improve, the degree of crystallinity does not often significantly increase. This suggests that the filler surface chemistry, loading, size, crystal nucleation, and chain mobility are all competing mechanisms in improving the crystallinity. Therefore, aside from the inclusion of a stronger filler and the expected constrained chain mobility, is the crystal structure itself within PEEK being positively influenced by the fillers?

In the case of GNP and CNT, the crystallinity remains the same or decreases with GNPs^10,16,21^ but is dependent on the loading and dispersion state with CNTs.^17,18,20−22^ These results suggest that carbon-based nanomaterials can stabilize PEEK on their surfaces via π interactions, but this same mechanism may also hinder further crystallization by impeding the chain mobility, corroborated by our previous work with molecular dynamics.^22^ In the case of ceramics, the PEEK crystallinity only increases at lower loadings, i.e., <1%, but decreases at higher rates at higher loadings compared to CNT/GNPs.^10,13,21,23^ which suggests that the differences in interface interactions and shape directly influence the chain mobility and crystallization.

Few studies of note have provided insights into how the structure and morphology of a secondary phase impact the crystal formation in PEEK.^10,24,25^ Available studies suggest that lattice matching and surface periodicity of the filler affects flat-on vs edge-on lamellar growth, and improved interface chemical interactions promote nucleation but can hinder the local chain mobility. In combination with varying cooling rates, the difficulty of controlling and characterizing the crystalline microstructure can quickly escalate. The lack of a consensus within the literature in the methodology for controlling the crystallinity in PEEK with nanofillers suggests that analyzing the crystalline architecture and developing a multifiller system is key in maximizing the mechanical behavior of the nanocomposites. Yet, little work has been done to fully validate this approach with visible trade-offs in the crystallinity and mechanical performance,^14,26^ but these studies suggest that finding the correct filler system might be the key in improving both metrics unilaterally.

Another problem with incorporating nanomaterials is controlling their dispersion. The high chemical stability of PEEK typically requires the use of concentrated acids for complete dissolution at low temperatures,^27,28^ such that conventional approaches of dispersion predominantly rely on thorough melt-compounding. The process of sulfuric acid dissolution/treatment results in direct sulfonation of the aromatic backbone, improving the ion conductivity and hydrophilicity, but drastically reduces the thermal stability of PEEK and complicates the mechanical behavior because the sulfonate group can both plasticize and stiffen PEEK according to the degree of sulfonation.^29^ Noncovalent dispersion methods are necessary if the goal is to avoid compromising PEEK’s existing properties and the nanomaterial’s functional properties, while improving the manufacturing scalability. A method of note involves the use of functionalized clays—sodium montmorillonite (MMT)—as a codispersant for PEEK/CNT nanocomposites.^26,30,31^ Negatively charged colloid–colloid interactions can drive codispersion of CNT/clay in water^32^ and within hydrophobic polymers^26,30,31,33,34^ to improve the mechanical and functional properties of PEEK nanocomposites. However, these studies suggest that MMT’s reinforcement ability is strongly dependent on the filler loading and exfoliation/intercalation of MMT with the matrix and filler. As a codispersant, MMT shows promise at improving the crystallization behavior only at low concentrations,^26,33,34^ which raises the following questions: how do filler systems synergize to improve crystallization behavior and do other alternative materials exist that could serve a similar or better function for PEEK nanocomposites?

To first address the dispersion of the nanomaterials, we apply a novel manufacturing methodology using nanocellulose to fabricate PEEK-GNP nanocomposites from a direct powder charge, tackling both noncovalent dispersion of hydrophobic nanomaterials into PEEK and nanomaterial-assisted modification of the crystallization kinetics. Here, the powder charge is comprised of PEEK powder, cellulose nanocrystals (CNCs), and GNPs ultrasonicated in water and then dried. CNCs are employed as a binder to adhere hydrophobic nanomaterials to the hydrophobic surfaces of micron-sized PEEK powder before processing. CNCs, which consist of polymerized glucose molecules, are the most abundant natural polymers available, with tensile strengths of 7.5–7.7 GPa, while containing abundant surface −OH groups for hydrogen bonding or chemical functionalization.^35,36^ CNCs are shown to exhibit both hydrophilic and hydrophobic properties according to the crystal facet exposing −OH groups and the degree of esterification introduced during the synthesis via acid hydrolysis^35,36^ easing dispersion of the the hydrophobic nanomaterials in hydrophilic environments.^37^ With the assisted use of ultrasonication, we have shown that the effectiveness of dispersing carbon nanomaterials can be attributed to covalent bonding between CNCs and defect sites in graphitic nanomaterials.^38^ The use of CNC as an effective reinforcing agent is widespread in its use across polymer nanocomposites^36,39,40^ and fiber-reinforced composites,^38,41−43^ but the CNC hydrophilicity has naturally led to works focusing on hydrophilic polymers with little attention on direct dispersion in aromatic, hydrophobic polymers like polystyrene^44^ due to agglomeration and dispersion issues.

Given the distinct chemical structures and shapes of CNCs and GNPs, this study aims to deepen our understanding of how the filler structure and shape influence the crystallization behavior in polymers. While existing literature has highlighted the significant role of the filler size on crystallization kinetics, the specific impact of the fillers’ structures on the nucleation behavior requires further study. CNCs, for instance, may not significantly interact with PEEK’s ketone and ether linkages due to the lack of strong dipoles like those found in the −OH groups with CNC. Consequently, we anticipate that CNCs primarily interact with PEEK through hydrophobic interactions via the crystal facets without exposed −OH groups. This interaction facilitates the coating of solid PEEK powders with CNCs, but it is unclear how these interactions influence the PEEK’s crystallization behavior during the melt state because no studies have directly investigated CNC-PEEK nanocomposites. On the other hand, the interaction between GNPs and PEEK can be more concretely hypothesized based on existing studies with carbon-based fillers like fibers, CNTs, and GNPs. These studies suggest that the nucleation behavior is influenced by the surface structure of graphite, which facilitates π-stacking interactions between PEEK and the graphite basal planes.^22,45,46^ This phenomenon is relatively well-studied, yet the specific alterations in PEEK’s crystallization behavior resulting from these π-stacking interactions remain unclear.

While the general influence of carbon-based fillers on PEEK’s crystallization is understood, pinpointing the exact changes in PEEK’s behavior due to these interactions requires further investigation. Recognizing that crystallization is hindered in fast-cooling rates, this study aims to investigate the central hypothesis of whether a designed spatial geometry achieved through a hybrid nanomaterial system, i.e., CNC:GNP, can be used to favorably mosaic and tailor the crystalline morphology and architecture to counteract the lower degrees of crystallinity from fast cooling and thus maintain the mechanical performance obtained from a slow-cooling process. We focus on analyzing the structure–process relationship between the cooling rate, nanomaterial morphology, and crystalline structure to provide a scalable manufacturing methodology for creating PEEK nanocomposites with enhanced structural properties. To evaluate the impact of the manufacturing rate, we produced panels through cooling the molds at two distinct cooling rates, slow (via natural convection, −0.7 °C/min) and fast (via water-assisted quenching, −460 °C/min). Our study finds that CNCs and GNPs both alter the crystalline morphology of PEEK at these extreme cooling rate variations, despite differences in structure and chemical composition. Interestingly, our results reveal that CNCs have a pronounced influence on the spherulite morphology and mechanical performance despite expected degradation, suggesting that lattice matching between CNC and PEEK lamellae is a promising approach to controlling the crystal architecture. Our findings highlight that a combined CNC:GNP system is a scalable method for dispersion and a viable method for microstructural engineering. Both CNC and CNC:GNP systems can counteract the low stiffness and strength attributed to low crystallinity from fast cooling, by either modifying the crystalline structure or introducing a stiffer filler. By qualitatively assessing the crystalline structure and the resulting tensile and impact properties, this study highlights the intricacies between the cooling rate, filler composition, crystallization kinetics, and the mechanical response of PEEK.

## 2 Methodology

### 2.1 Materials

PEEK powders (particle size: 50 μm) were used as received (Victrex PEEK 450PF). GNPs (EG016) with 2–5 μm dimensions were supplied from Celtig LLC. The CNC used was NCV-100 (CelluForce, Quebec, Canada) with a diameter of 2.3–4.5 nm and a length of 44–108 nm. The nanocomposite sample compositions and respective weight fractions of the additive nanoparticles for the concentrations are shown in Table 1. For this study, the nanocomposite samples contain a total of 1 wt % filler content by mass, which was found to be the optimum concentration for increasing the mechanical properties of the material without embrittlement from agglomeration or significant restriction in the chain mobility.^47^ The sample concentration corresponds to the final composition according to the weight fraction (*W*_F_) of the nanocomposite. GNP alone is not directly used in this study because pristine graphitic nanomaterials are not dispersible in water. Here, GNPs are combined with trace amounts of CNC (ratio of 1:12 CNC:GNP) to use the minimum CNC for dispersion of GNPs in water and to minimize the influence of CNC on the crystalline structure generated by GNPs. However, PEEK is also hydrophobic; therefore, during melt consolidation, hydrophobic interactions are required between PEEK and GNP for effective dispersion. Previous work demonstrates through water contact and ζ-potential measurements that the 1:12 CNC:GNP colloids are predominantly hydrophobic while remaining stable in water,^48^ thereby allowing us to coat PEEK powders with GNPs using water, before homogeneously melt-dispersing during compression molding. Additional details on the dispersion process are provided in Section S1.1.

**Table 1:** PEEK Nanocomposite Material Compositions

composition	filler content (% by mass)
Neat	100% PEEK
CNC	99% PEEK, 1% CNC
CNC:GNP	99% PEEK, 0.083% CNC, 0.917% GNP

### 2.2 Materials Processing

The nanocomposite powder charges were prepared using a wet mixing technique. For the PEEK-CNC panel, CNC was directly dispersed in 750 mL of deionized (DI) water using probe sonication (QSonica Q500 with a 1-in.-diameter probe) at 20 kHz, 75% intensity for 1 h in an ice bath. PEEK powders were then added to the suspension and mixed by magnetic stirring for several hours until no PEEK was floating on top of the suspension. Afterward, the suspension was placed on a hot plate and stirred until the water was fully evaporated. Finally, the powders were dried for 24 h at 100 °C under vacuum. The steps in the PEEK-CNC/GNP composite panel are the same as those in the PEEK-CNC panel, except GNP was added to the suspension after CNC was homogeneously dispersed in DI water. GNP was added to the CNC suspension and sonicated for an additional 1 h before PEEK powder was added. For the PEEK-only panels (labeled as Neat), PEEK powders were prepared and vacuum-dried according to the same procedure before direct processing into panels. Additional information regarding the preparation for the powders is provided in Section S1.1.

### 2.3 Molding Process

**Figure 1:**
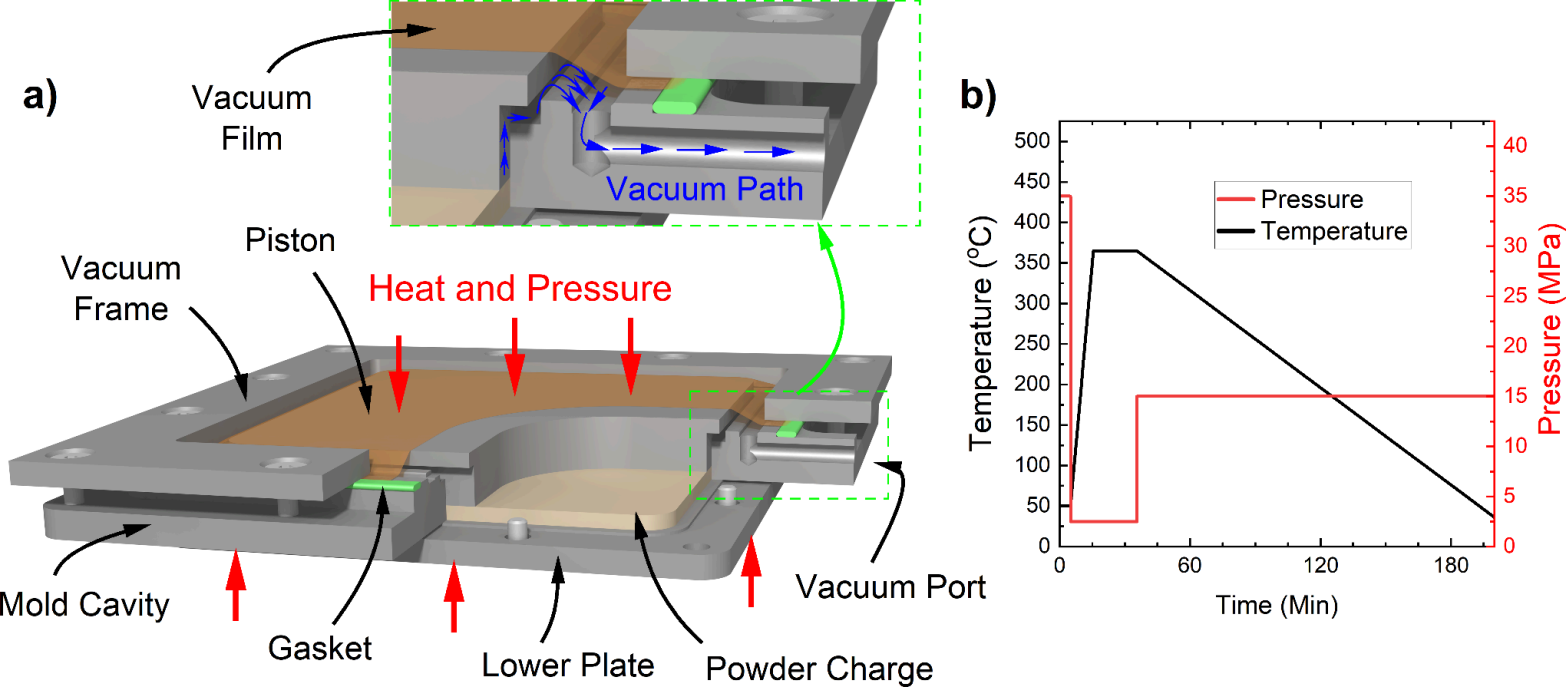
(a) Cross-sectional schematic view of the vacuum-assisted compression mold. Powders are loaded into the center of the mold before inserting the piston and then sealing with the vacuum film. The entire mold is placed in the hot press, where force is directly applied to the piston surface and lower plate during consolidation. (b) Consolidation cycle of the composite panels, indicating processing pressures and temperatures.

In this study, compression molding was used to fabricate the nanocomposite panels. Figure 1a shows a schematic view of the compression mold assembly. After loading the powders into the center cavity, the piston was inserted. A silicone-based sealant tape was used as a gasket in the cavity margins to hermetically seal the mold with a polyimide film. Finally, the vacuum frame was bolted down to the cylinder to ensure gases only pass through the vacuum port. After loading of the powders, the compression mold was placed into a 30-ton Carver automatic hot press (Autofour/3015-PL H 4533) to follow the consolidation cycle outlined in Figure 1b. During consolidation, the piston seals the mold, the heat melts the materials, and pressure brings the material into contact with all mold surfaces. The vacuum line removes any trapped moisture or gases until the molding material cools to room temperature. The combination of high compression force and vacuum minimizes the porosity. At the start of the consolidation cycle, 40 MPa of pressure at room temperature was first applied to the mold to fully pack the powders. Afterward, pressure was removed and the mold was uniformly heated to the melting point of PEEK. The mold was then held at a constant temperature for 20 min to create homogeneity within the melt. The platens’ heating was then turned off, the pressure was raised to 15 MPa, and the system was allowed to cool to room temperature by natural convection, ∼0.7 °C/min, to maximize the crystallinity.

**Figure 2:**
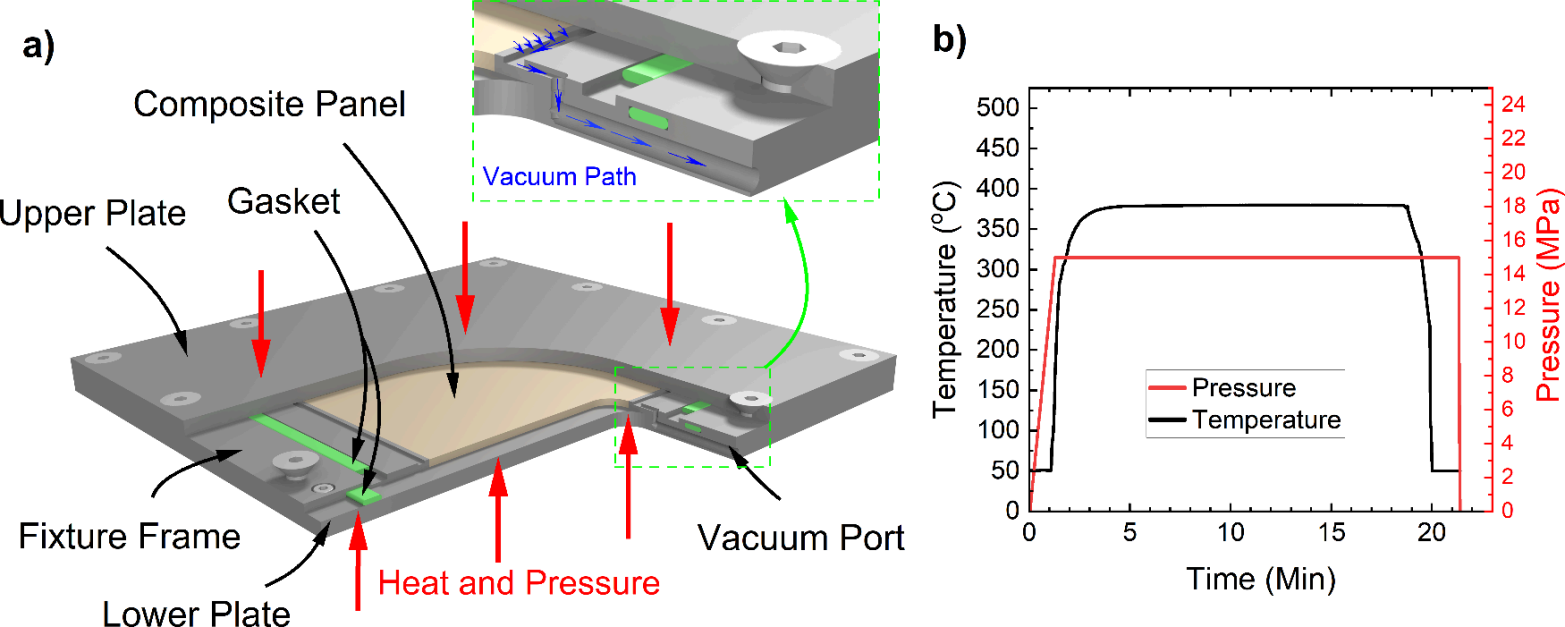
(a) Cross-sectional schematic view of the vacuum-assisted annealing mold. The panel is inserted into the mold, and the mold is fully assembled before placement in the press. Heat and pressure from the hot press are applied directly to the flat surfaces of the mold upper and lower plates. (b) Annealing cycle of the composite panels, indicating measured processing pressures and temperatures.

Due to the limited cooling capabilities of the hot press, a separate annealing fixture was manufactured for cooling the tooling at the desired high cooling rate. The composite powder charge was first molded into panels using the compression mold. Then the panels were reheated with the hot press inside the annealing fixture. Finally, the fixture is removed from the hot press and quenched in water. The schematic view of the annealing fixture is shown in Figure 2a. The fixture consists of three main parts: fixture frame, upper plate, and lower plate. The fixture frame was used to prevent lateral expansion and deformation of the panels when the temperature increases to the melting point of the composite panels. The upper and lower plates provide a flat, rigid surface to minimize warping during quenching. The two plates were of equal thickness for uniform heat transfer. Like the compression mold, the annealing fixture also had a port to apply a vacuum during the annealing process. The pressure and temperature history of the panels in the annealing process is shown in Figure 2a. The assigned naming convention for all nanocomposite specimens and their associated cooling rates are summarized in Table 2. Additional information regarding the molding and annealing process is provided in Sections S1.2 and S1.3.

**Table 2:** Specimen Naming Convention and Corresponding Cooling Rates^a^

specimen ID	cooling rate
Neat-Fast	460 °C/min
CNC-Fast	
CNC:GNP-Fast	
Neat-Slow	0.7 °C/min
CNC-Slow	
CNC:GNP-Slow	

a All compositions contain PEEK, whereas Neat refers to PEEK without fillers.

### 2.4 Characterization Techniques

**Figure 3:**
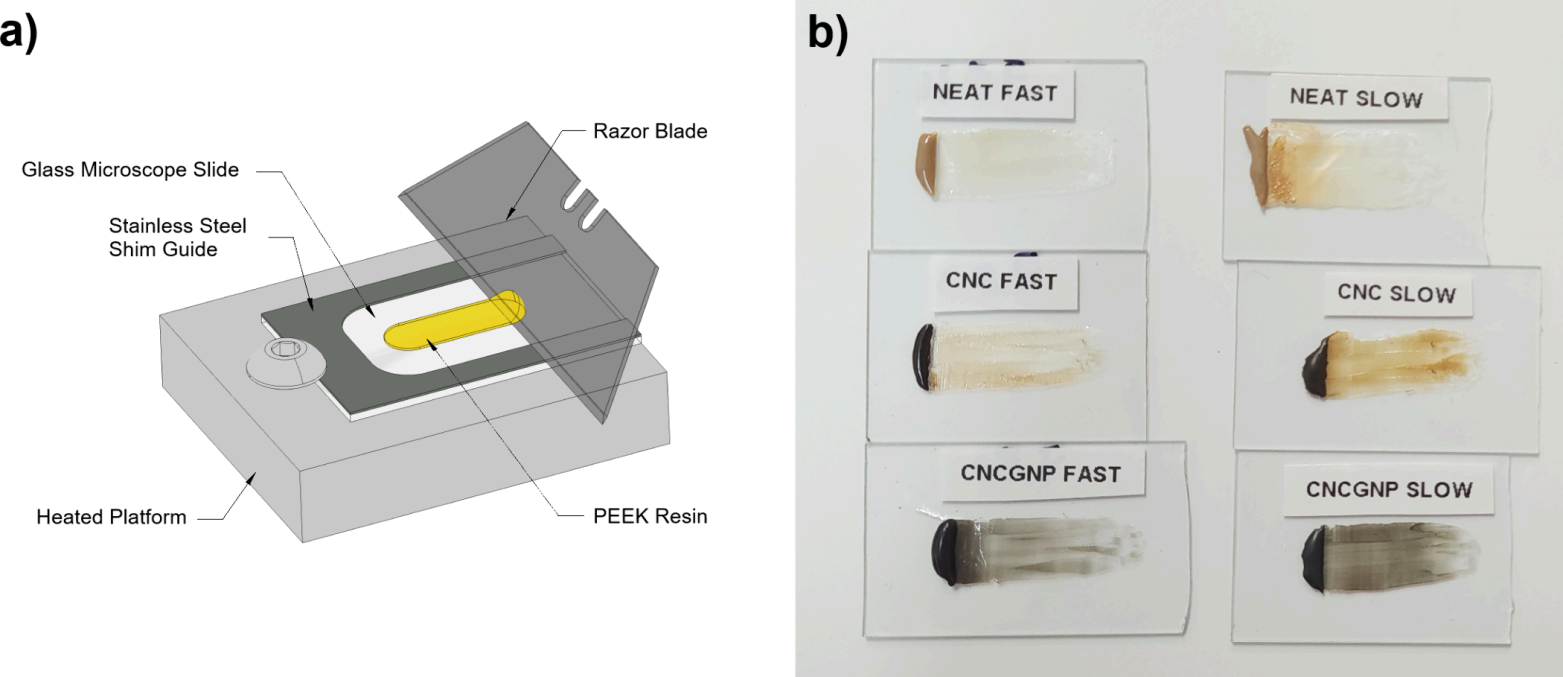
Manufacturing process of PEEK thin films: (a) PEEK thin-film fabrication fixture; (b) thin-film samples after manufacturing and cooling in platens.

Polarized optical microscopy of PEEK thin films was performed using a benchtop Olympus CH-2 optical microscope with a 100× oil immersion lens. For maximum contrast of the crystalline microstructure, the specimen was placed under linear cross polars at 90°. To fabricate the specimens for optical microscopy, a doctor blade methodology was employed. Figure 3a demonstrates the fixture used to create the films, in which a bead of sample resin (∼10 mg) is melted on a glass slide placed on a platform heated to 360 °C. Temperature was slightly above the melting point of PEEK of 343 °C to ensure a liquid state but to minimize oxidation at higher temperatures. A razor blade coated with surface sealant B-15 and polymer release agent Frekote 700-NC was then heated to the temperature of the platform. The resin bead is then scraped along the surface of the glass slide using 50 μm stainless steel shims stock as a thickness guide. Films may contain parallel grooves associated with uneven sharpness of the blade. Therefore, some regions have a thickness <50 μm. Glass slides containing the thin films were placed on the heated platens surface set to the molding temperatures (Figures 1a and 2a) and subsequently cooled using the same rates for the panels. The final films after reheating and cooling are shown in Figure 3b.

X-ray diffraction (XRD) patterns were recorded using a Rigaku Miniflex 600 X-ray diffractometer. X-rays (1.54 Å from a Cu anode) are generated using 40 kV and 15 mA. Disk samples of 20 mm diameter were cut from the panels. The surfaces of disk specimens were abraded to remove the polymer that contacted the mold surface. Samples were scanned from 3 to 90°, with a 0.01° step size and a 10°/min scan rate. The samples were rotated and rescanned such that the diffraction spectra shown correspond to the average of at least three scans across random directions. For the pure GNP spectra, powder samples were directly tested. For pure CNC, a 2 wt % CNC–DI water solution was prepared and evaporated onto a glass substrate to create films that mimic the CNC coatings from the PEEK powder charges. The interplanar spacing (*d*-spacing) is calculated using Bragg’s law and the scattering angle.
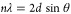
1

The crystal coherence/size (*L*) along the crystal plane (*hkl*) was calculated using the Scherrer equation:
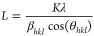
2where *K* is the Scherrer constant set to 1,^49^ λ is the X-ray wavelength of 1.54 Å, β is the full width at half-maximum (fwhm) of the diffraction peak, and θ is the Bragg angle.

Small-angle X-ray scattering (SAXS) patterns were recorded using using a Xenocs Xeuss 3.0 system. Similarly to the XRD, disk samples were used, which were scanned with an exposure time of 120 min. The long period, defined as the combined crystalline and amorphous region which composes each individual crystallite within the nanocomposite, was calculated using the Lorentz-corrected SAXS curve and SASAnalysis software.

Differential scanning calorimetry (DSC; Netzch DSC214 Polyma) was used to determine the degree of crystallinity amount (*X*_c_) of the samples. Samples of 7–10 mg were prepared by slicing fragments along the thickness of each panel. Samples were placed in a nonhermetically sealed pan with a pinhole and then heated to 450 °C at 10 °C/min under nitrogen. *X*_c_ was calculated using
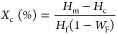
3where the cold crystallization enthalpy (*H*_c_) and heat of melting (*H*_m_), were determined by integrating the area of the first and second peaks observed in the heating curve, respectively. The enthalpy for pure crystalline PEEK (*H*_f_) was assumed to be 130 J/g,^50^ and the weight fraction of fillers (*W*_F_) is 0 for Neat (PEEK only) and 0.01 for PEEK-CNC (labeled as CNC) and PEEK-CNC:GNP (labeled as CNC:GNP).

The ASTM D638-14 standard test method for the tensile properties of plastics was used to measure the low-strain rate tensile properties of composites, i.e., tensile strength, elastic modulus, toughness (stress–strain curve area), and failure strain. Based on the limited thickness of the panels (3.2 mm), the type V specimen was chosen and tested at a displacement rate of 10 mm/min. For the purpose of characterizing the high strain rate behavior of the nanocomposites, the Izod impact test according to ASTM D256 was used. Standard V-notched specimens measuring 63.5 mm × 10.5 mm × 3.2 mm with a 45° notch of tip radius of 0.25 mm were machined from the panels for this experiment.

## 3 Results and Discussion

### 3.1 Polarized Optical Microscopy

**Figure 4:**
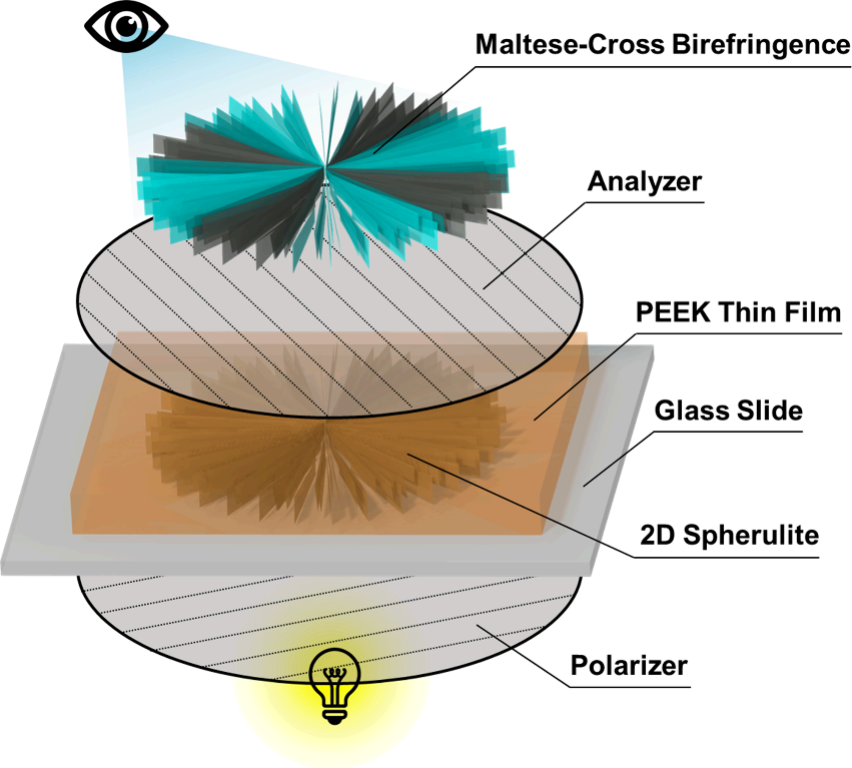
Polarized optical microscopy schematic of the Maltese-cross patterns observed from PEEK 2D thin-film spherulites.

**Figure 5:**
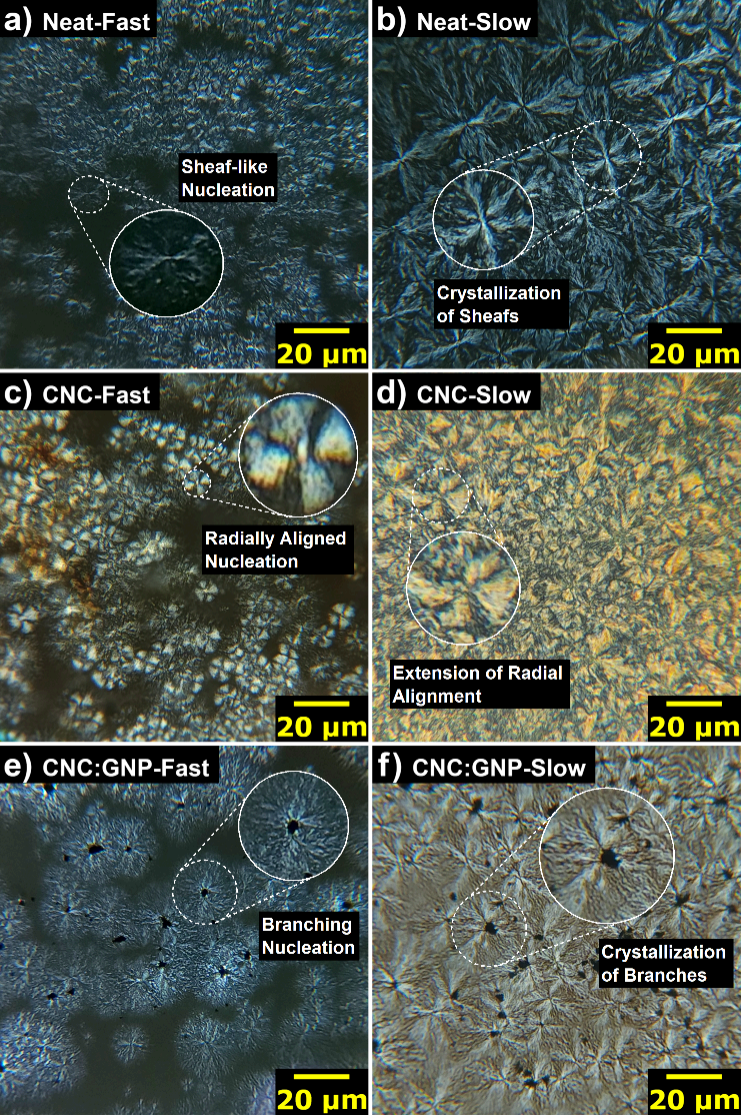
Polarized optical microscopy of PEEK nanocomposite thin-film spherulites: (a) Neat-Fast; (b) Neat-Slow; (c) CNC-Fast; (d) CNC-Slow, (e) CNC:GNP-Fast; (d) CNC:GNP-Slow. Each image has a spherulite highlighted and morphology described for comparing the changes in structure.

Polarized optical microscopy reveals microstructural changes due to the inclusion of CNC and CNC:GNP systems within PEEK. Figure 4 shows that the Maltese-cross patterns, indicative of spherulitic morphology within PEEK, appear when 2D cylindrical spherulites diffract polarized light aligned with the direction of cross polars. These patterns, observed in our specimens from Figure 5, corroborate prior findings that PEEK thin-film spherulites typically exhibit cylindrical morphology.^51^ Although the formation of 2D spherulites is assisted by the effects of confined crystallization of the thin films,^52^ our results highlight that the change in the cooling rate and the inclusion of nanomaterials influence the nucleation mechanism and spherulite morphology. In Figure 5, polarized micrographs of the PEEK nanocomposite thin-film specimens depict the crystalline microstructure within these samples. Polarized light passing through amorphous regions does not diffract, resulting in large dark regions after passing through the analyzer. The brightest regions mark the crystalline areas aligned with the cross polars: orange tint indicates CNC agglomerates (Figure 5c), and black markings are GNPs (Figure 5e,f). Note the dark regions within the spherulites are either amorphous or crystalline regions off-axis from the cross polars, depending on the structure of the spherulite.

In fast-cooled specimens, the influence of the filler composition on the nucleation process is pronounced. Unlike Neat-Fast and CNC:GNP-Fast, where spherulite nucleation is observed to be sheaf-like from a principal direction or highly branched, respectively, CNC-F demonstrates dense, radially symmetric nucleation (Figure 5a,c,e). Despite the functionalization-like effect of CNC on GNPs (Figure 5e), the crystals around GNPs lack the Maltese-cross patterns observed in the other compositions. Very few regions of the GNP surfaces can contain CNC-mediated interface interactions, as shown by previous SEM images, where CNCs attach in small clusters to the GNP basal planes.^38^ The highly branched morphology of CNC:GNP is likely attributed to edge-on nucleation on GNP surfaces (supported by the microscopy of PEEK-pure GNP in Figure S9) and nucleation competing with surface CNCs. These observations on the relationship between the nanomaterial composition and morphology suggest a direct correlation between the branching morphology and spherulite size. The order from smallest to largest observed size, which mirrors the morphology from least to most branched, is CNC, Neat, and CNC:GNP. This indicates that the spherulite size and morphology can be directly manipulated by the morphology of the nanomaterials.

The effect of a slower cooling rate is common across all three specimens, and spherulites are given further time to grow and restructure. In the case of CNC (Figure 5d), spherulites that began radially aligned maintain the same alignment while crystallizing the remaining amorphous content. The result is many scattered spherulites with small dendritic structures. The general lack of spherulite size homogeneity may be due to the competing effects of limited chain mobility induced by the size of the CNC and the preferential nucleation on CNC agglomerates. For Neat and CNC:GNP specimens (Figure 5b,f), the slower cooling rate allows the initial sheaf-like and branched spherulites, respectively, to further expand and crystallize.

The general size and morphology of the spherulites in these two specimens are similar in slow cooling, suggesting that GNPs have a limited negative influence on homogeneous crystallization compared to pure CNCs.

**Figure 6:**
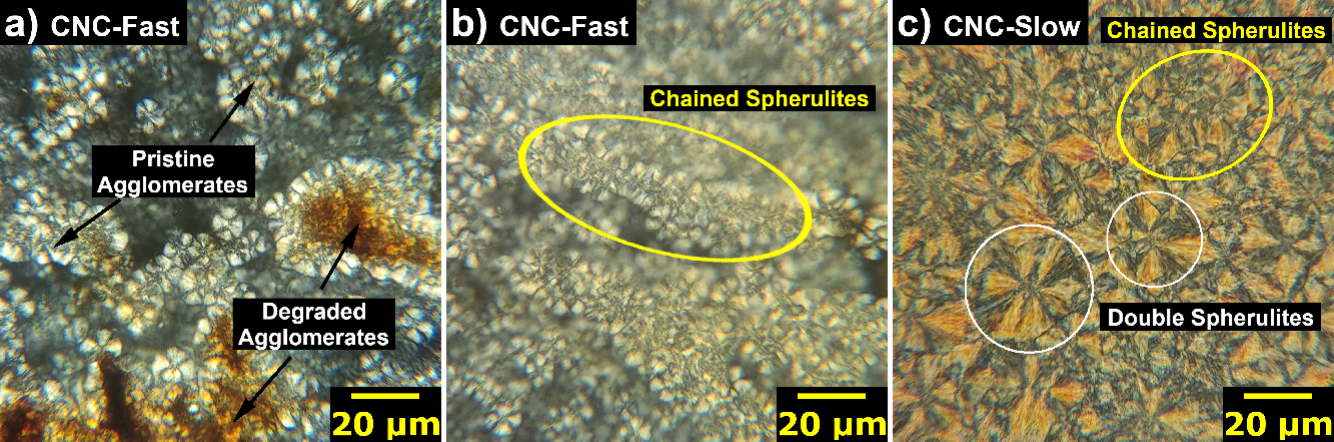
Micrographs of PEEK-CNC demonstrating the effect of nucleation and miscibility. (a) Large CNC agglomerates in fast cooling. Degradation of CNC is observed by the change in color to orange. (b) CNC agglomerates in fibril formations, demonstrating the ability to form chains of PEEK spherulites. (c) Presence of the chained spherulites showing the persistence after slow cooling.

In Figure 6a, we observe significant spherulitic growth near the orange-tinted agglomerates in the CNC specimens, highlighting CNCs’ ability to directly promote nucleation. The orange color of these agglomerates is an artifact of thermal degradation, which occurs at temperatures above 240 °C. At these temperatures, cellulose is expected to undergo chain scission as glycosidic bonds cleave, forming levoglucosan, organic vapors, and other volatiles.^53^ Parts a and b of Figure 6 show radial, heterogeneous nucleation stemming from dispersed CNCs, with Figure 6b specifically showing linearly connected CNC agglomerates producing chained spherulites. Thermally decomposed CNCs show miscibility in the PEEK melt serving as nucleation sites while creating a highly ordered radial lamellar structure of the spherulites. This is evident in Figure 6c, where large spherulites contain orange patches on the spherulite “leaves”, still maintaining the radially symmetric structure from fast cooling.

Chen and Yang showed that miscibility can impact the crystalline structure through their study with different ratio PEEK/PEKK blends.^54^ Here the quantity and miscibility of the secondary polymer can directly affect the spherulitic structure, indicating in-plane bending or twisting of the lamellar structure. In our study, however, we found that CNC’s directly compliment the spherulitic structure of PEEK given that no such banded changes in the birefringence pattern of the slow-cooled Maltese-cross patterns are observed. Interestingly, in key locations, spherulites can fully nucleate side-by-side if they share a sufficiently large CNC content (circled in white in Figure 6c). In regions with linear formations, as seen in Figure 6b, chained spherulites form that compete in creating a uniform structure (circled yellow in Figure 6c). Based on these observations, the use of CNC agglomerates or larger-scale cellulose fillers can be strategically used to introduce anisotropy to the crystalline structure of PEEK.

In general, our results support the notion that nanomaterials can influence the crystallization kinetics,^11,16^ yet the specific factors or properties dictating this behavior are unclear without further analysis. While both CNCs and GNPs directly impact nucleation, thereby ruling out size as a factor, it becomes necessary to determine if surface chemistry, surface topology, or physical properties (such as thermal conductivity) must be considered.

#### 3.1.1 Discussion of the Filler’s Influence on Crystallization

Chen and Hsiao previously studied whether the surface free energy, which is influenced by surface chemistry or surface topology, influenced edge-on or flat-on nucleation on carbon fiber surfaces. They evaluated this theory through a qualitative study involving pitch and PAN-based carbon fibers, in combination with aramid-based sizing, plasma treatments, and different high-performance aromatic thermoplastics (PEEK, PEKK, and PPS).^46^ They find that surface nucleation is not dependent on surface chemistry because plasma treatment of carbon fibers did not affect the development of the transcrystalline region. Rather, the type of carbon fiber (pitch vs PAN) and the use of an aramid sizing agents with similar unit cell structure dictated surface crystal nucleation. Because changes in surface chemistry does not influence interface crystallization in their study, they suggest localized undercooling due to the high thermal conductivity of fibers, and interactions between PEEK and the structure of graphite at the fiber surface may play crucial roles.^46^

We cannot completely rule out surface chemistry. PEEK is hydrophobic due to its aromatic structure, but the ether and ketones still have weak dipole moments likely capable of interacting with the hydroxyl and ether groups in cellulose and levoglucosan (primary decomposition product of cellulose). The edge sites in GNPs would also contain similar oxygen functional groups, and we should also consider π-stacking interactions between PEEK and GNP basal planes. Despite these expected differences in van der Waals (vdW) interactions, we visually confirm that both CNCs and GNPs are capable of nucleating PEEK. Surface chemistry must still be considered because, at the very least, it is a key factor in ensuring wetting of the fillers.

Chen and Hsiao’s next hypothesis of crystallization via undercooling is logical, suggesting that a thermal conductivity mismatch promotes preferential nucleation on high-thermal-conductivity fillers capable of dissipating heat. However, this scenario is unlikely in our study because the thermal conductivity of bulk CNC films (0.22–0.53 W m^–1^ K^–1^ according to directionality)^55^ is comparable to PEEK’s bulk thermal conductivity (0.24 W m^–1^ K^–1^).^56^ Despite this, CNCs clearly influence crystallization compared to GNPs, which have higher thermal conductivities by several orders of magnitude.

Therefore, the only remaining hypothesis to consider is surface topology (interactions due to the compatibility of the graphite structure with PEEK), which suggests that some degree of lattice structure compatibility influences the nucleation behavior. This conclusion aligns with the literature observations of PEEK transcrystallinity when using pitch versus PAN carbon fibers. The differences in PEEK’s nucleation ability appears to correlate with the differences in exposed defect sites, edges, and basal planes between pitch and PAN carbon fibers.^6^ Supporting this, a previous study where epitaxial growth of PEEK on graphitic and silicone/stainless steel substrates was evaluated proposed that edge-on nucleation is dependent on lattice matching between polymer chains and the substrate.^24^ To explain the nucleation mechanism of PEEK on graphite, a previous work that simulated PEEK–CNT interactions via molecular dynamics indicates that π-stacking interactions between PEEK and CNT result in PEEK chains stabilized in a planar orientation along the CNT surface.^22^ This supports the notion that surface interactions dictate edge-on nucleation, which is verified by the edge-on nucleation observed in a PEEK-pure GNP nanocomposite that we prepared via direct melt-compounding (Figure S9). TEM micrographs from the literature on PEEK-graphene nanocomposites also seem to support this observation of edge-on nucleation on basal planes.^15^

In summary, our microscopy studies indicate that a CNC’s primary role of improving crystallization can be attributed to its small size and some presumable lattice stabilization that requires further crystallographic analysis (discussed in Section 3.2). When CNC is paired with GNPs (1:12 ratio by mass), nucleation on the GNP surfaces still favors edge-on nucleation (Figure 5f). A previous investigation on molecular interactions between CNC and carbon nanomaterials states that CNCs can be oriented flat to maximize vdW interactions or standing when ultrasonication induces covalent bonding on defect sites.^57^ It is plausible that CNCs promote edge-on nucleation in both orientations, but this requires further analysis into the crystal structures of the nanocomposites to conclusively decide on a growth mechanism. Contrary to the initial assumption that the CNC’s hydrophilicity and thermal degradation only adversely affect the nanocomposite, CNC visibly introduces order to the crystalline structure in addition to its vital role in dispersing and stabilizing hydrophobic nanomaterials (GNP here) using water-based methods.

### 3.2 DSC

The degree of crystallinity and bulk crystallization kinetics of the specimens were assessed by examining the differences in the DSC heating traces. Although XRD and wide-angle X-ray scattering (WAXS) are limited in measuring the crystallinity according to the preferential alignment effects observed (Figures S10 and S11), DSC allows us to calculate the bulk degree of crystallinity (*X*_c_) from the manufacturing process via sampling shavings across the thickness. The inclusion of either CNC or CNC:GNP notably affects the enthalpy at the glass transition temperature (*T*_g_), the secondary crystallization behavior, and the bulk degree of crystallinity.

**Figure 7:**
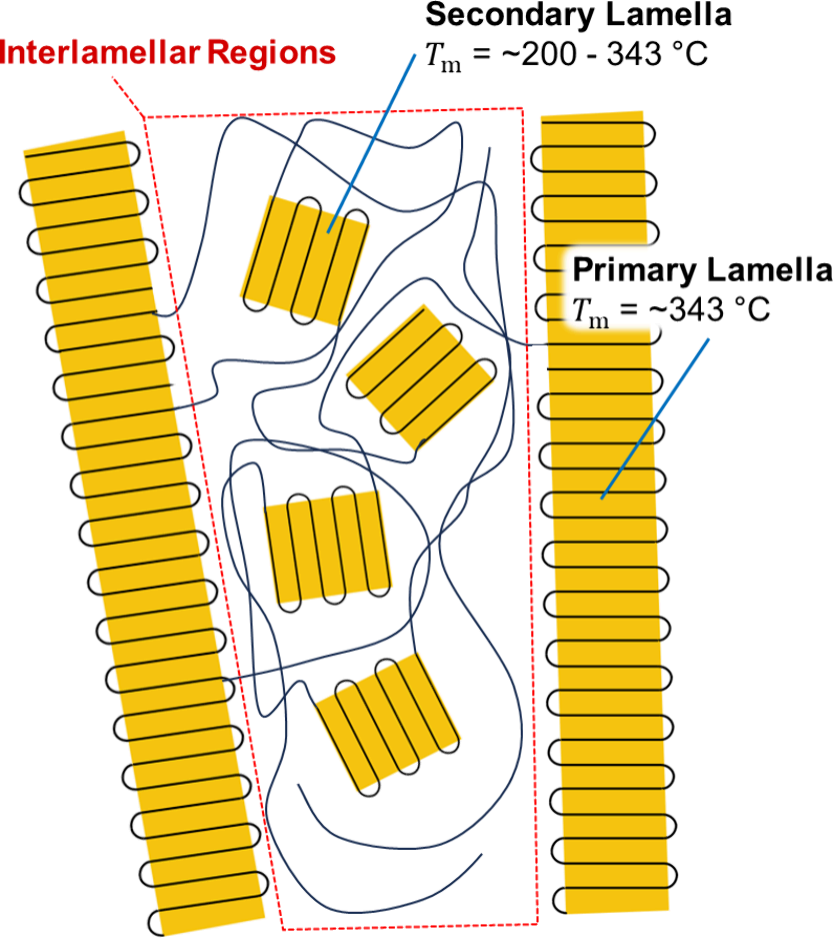
Schematic of the secondary crystallization behavior associated with the double melting observed in DSC scans of PEEK.

**Figure 8:**
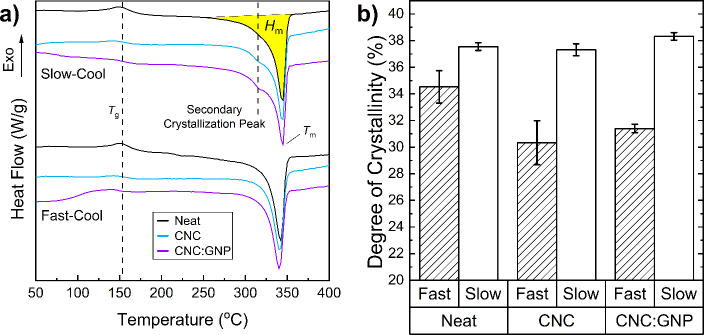
(a) DSC heating scans of fast- and slow-cooled nanocomposite samples. For reference, the locations of the *T*_g_, secondary crystallization, and melting peak regions used for the bulk degree of crystallinity calculations are highlighted. (b) Corresponding bulk degree of crystallinity calculated for each nanocomposite.

Historical works on the crystallization behavior of PEEK have indicated that two endothermic peaks can be observed within the primary melting endotherm due to the differing melting temperatures of the primary lamellae and the secondary interlamellar crystallites (Figure 7).^58,59^ Cold crystallization and secondary crystallization are distinct yet related phenomena. Cold crystallization occurs when highly amorphous regions restructure into more ordered, lower-energy states when heated. This process is typically identified by an exothermic peak during DSC with highly amorphous samples (Figure S12), indicating that cold crystallization is a scan-sensitive occurrence during measurement. Similarly, secondary crystallization focuses on the presence of interlamellar crystals (secondary lamellae) and a thickening of the primary lamellae. Additional crystalline structures are detected as an endothermic peak separate from the primary melting endotherm, whose magnitude and location has been shown to be influenced by the annealing temperature and time.^58,60^ The secondary crystallization behavior is analyzed according to the presence of cold crystallization and the changes in the minor peak within the melting endotherm (310 °C), primarily evident in the slow-cooled heating traces (Figure 8a). For reference, modulated DSC scans of fully amorphous PEEK were additionally recorded in Figure S12 to mark the location of the cold crystallization peak.

Here in Figure 8a, the DSC scan mirrors the reverse behavior of the crystallization history. The first signal near 150 °C corresponds to the observed heat flow from chain relaxation as the material exceeds *T*_g_. With continued heating, cold crystallization will demonstrate itself as an exothermic peak between *T*_g_ and *T*_m_ for PEEK, which is not observed across all specimens. As melting begins near 300 °C, the relative crystallinity gradually decreases due to the melting of lower-order crystallites from secondary crystallization. Finally, heating to the temperature of the highest magnitude endothermic peak corresponds to melting of the primary lamellae.

In both fast- and slow-cooled specimens, the inclusion of CNC and CNC:GNP reduces the enthalpy change at *T*_g_, indicating that CNC and CNC:GNP inhibit chain mobility. Across all samples, no cold crystallization is observed, pointing to the inability of the quenching process of the mold to internally disperse heat sufficiently to create a highly amorphous panel. The key distinction between fast- and slow-cooled scans, as observed in Figure 8a, lies in the secondary crystallization peak’s presence. During slow cooling, this peak is more pronounced with CNC inclusion and even more so with CNC:GNP, suggesting their role in promoting secondary crystallization. Conversely, in the fast-cooling process, the secondary crystallization peak does not appear, suggesting that the rapid cooling limited the formation of secondary crystallites.

From Figure 8b, CNC and CNC:GNP slightly change the degree of crystallinity (*X*_c_) of slow-cooled PEEK. According to the data in Table 3, the peak height of the melting endotherm decreases with the addition of CNC and further with CNC:GNP. This reduction in the primary endotherm’s peak intensity, coupled with the observed increase in crystallinity, implies that the main factor behind the slight increase in crystallinity is attributed to the secondary crystallization peak.

**Table 3:** DSC Results of Melting Endotherm Peak Heights, Melting Enthalpies, and Calculated Bulk Degrees of Crystallinity

composition	*H*_m_ peak height (W/g)	*H*_m_ (J/g)	*X*_c_ (%)
Neat-Fast	0.392	44.9	34.5
CNC-Fast	0.349	39.0	30.3
CNC:GNP-Fast	0.361	40.4	31.4
Neat-Slow	0.402	48.3	37.5
CNC-Slow	0.382	48.0	37.3
CNC:GNP-Slow	0.369	49.3	38.3

In the fast-cooled samples, the crystallinity with CNC and CNC:GNP is reduced by 12% and 9%, respectively, relative to that of Neat PEEK. This decrease in crystallinity for both samples is corroborated by the microscopy images (Figure 5a,c,e) because CNC-Fast spherulites are shown to be smaller than Neat-Fast spherulites, and CNC:GNPs branching morphology lacks visible interlamellar crystallization. The concentration of CNC must be carefully optimized to maximize crystal growth and nucleation without substantially impacting the overall chain mobility at rapid cooling rates. With annealing, CNCs will improve infilling and secondary crystallization, with trace amounts improving the secondary crystallization kinetics of the CNC:GNP system.

### 3.3 Crystallography

**Figure 9:**
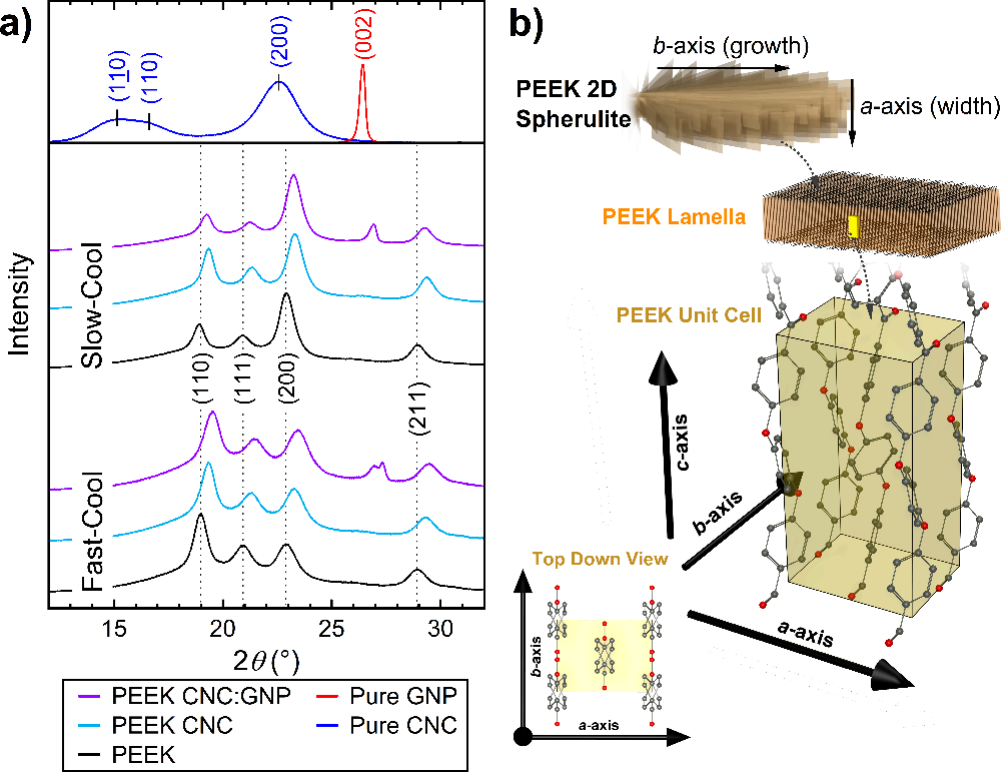
(a) Normalized XRD spectra for PEEK Neat/CNC/CNC:GNP nanocomposite samples. Spectra for pure CNC and GNP are included at the top for compositional references. Marked with corresponding colors are the locations of the key reflections of indices (*hkl*) associated with the crystalline structures of CNC, GNP, and PEEK. (b) Unit cell (*abc*) and index directions of PEEK lamellae according to refs (51) and (59) and expected growth directions of lamella in PEEK spherulites.

In Figure 9a, XRD patterns depict the relationship between the crystalline structure and specimen compositions considering different cooling rates. The specimens after molding were directly characterized to focus on the 3D spherulite structure, whereas the previous polarized microscopy results analyzed the 2D morphology. Lovinger and Davis^51,61^ previously proposed that PEEK’s 2D crystalline formation in thin films is dominated by uniplanar lamellar growth, irrespective of substrate epitaxy. They define that the *a* axis corresponds to the width of the lamella, the *b* axis to the lamellar preferred growth direction, and the *c* axis to the alignment direction in-plane with the lamellae (Figure 9b). Given these distinctions, we narrow the interpretation of our XRD results to changes in the lamellar structure across the cooling rates and compositions.

Black, light-blue, and orange peaks in Figure 9a mark the reflections from the primary diffraction planes within the PEEK nanocomposites. Following Lovinger and Davis, the PEEK unit cell’s *c* axis is aligned with the thickness direction of the lamella (Figure 9b). The differences between fast- and slow-cooling spectra indicate that the cooling rate primarily affects the relative intensities of the (110) and (200) reflections, which correlate with growth along the *b* and *a* axes, respectively. Notably, the inclusion of nanomaterials across both cooling rates results in a right shift in the spectra, suggesting lattice strain within the PEEK crystallites. For reference, Table S1 provides the scattering peak positions and fwhm of the peaks.

#### 3.3.1 Analysis of the Crystallite Size

**Figure 10:**
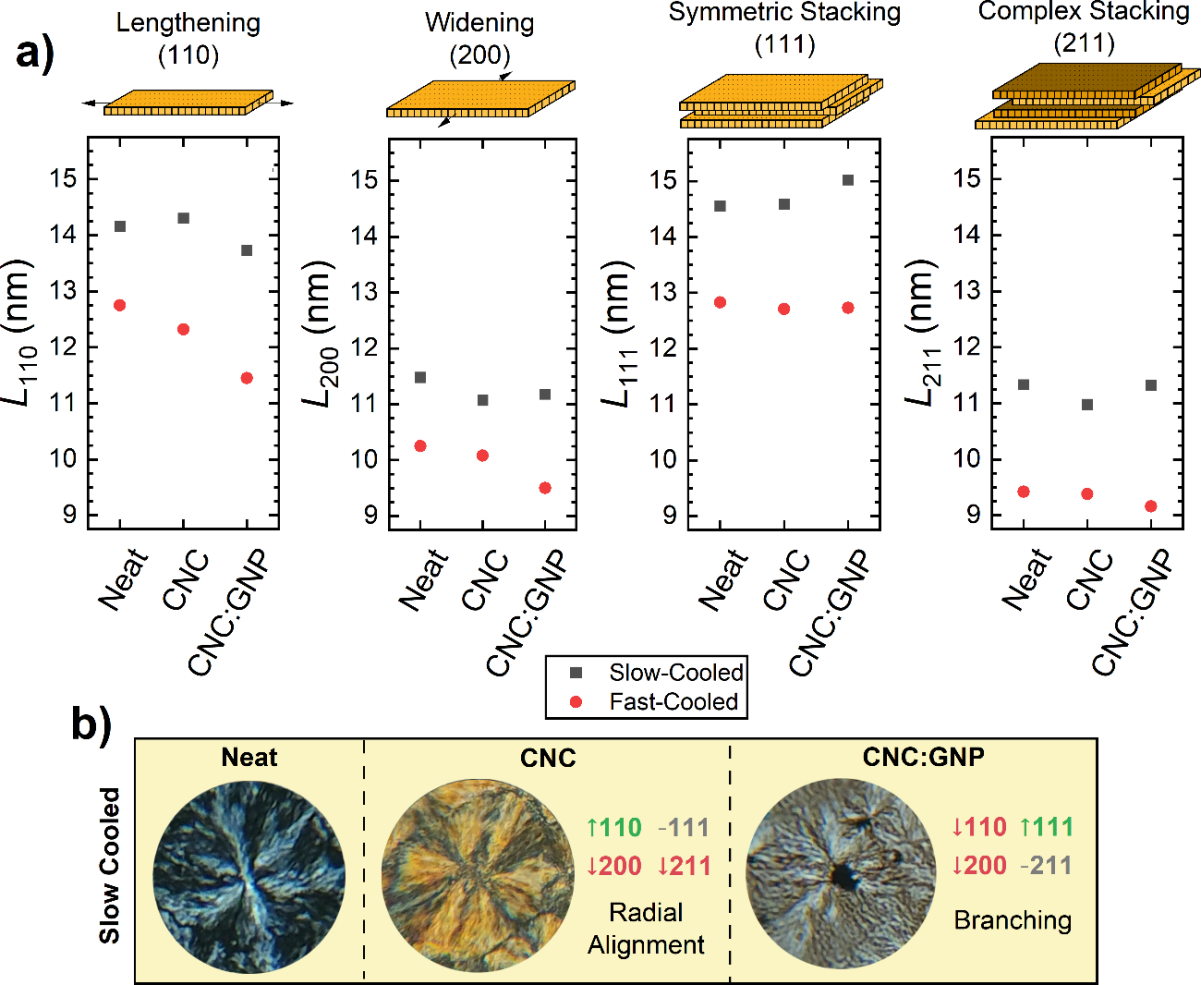
(a) Comparison of the crystallite size along the key diffraction planes of PEEK in slow versus fast cooling of the nanocomposites. (b) Correlation in the crystallize size changes to spherulite morphology. The correlation to slow is only shown because slow-cooled samples contain the least significant change in the degree of crystallinity, thereby isolating the changes in morphology to the changes in lamellar growth mechanisms.

In the fast-cooling process, the formation of primary lamella from the amorphous melt is expected; thus, the high intensity of the (110) reflection in Figure 9a aligns with the literature-defined lamellar growth direction. Conversely, in slow cooling where infilling and crystallization of the remaining amorphous regions is expected, the (200) reflection dominates the spectra, suggesting that the formation of crystals in the interlamellar regions or the widening of the principal lamellae drives further crystallization. In both cooling methods, reflections along the *c* axis [(111) and (211)] have a lower intensity than those along the *a* and *b* axes [(110) and (200)], indicating limited lamellar thickness or stacking of adjacent lamellae. This observation of weak *c*-axis reflections, corroborated by previous studies on acid-etched and solution-casted PEEK thin films,^58,61^ suggests that the bulk of the crystallinity stems from growth along the *a* and *b* axes. Figure 10a plots the changes in the crystallite size along the four key diffraction planes of PEEK. Here, we observe that the (110) and (111) planes typically exhibit higher crystallite sizes, suggesting that growth occurs along the lengthening plane (110) and lamellar stacking (111) direction. Conversely, the smaller crystallite sizes in the widening (200) and complex stacking (211) planes suggest that the crystallization mechanism is first initiated by lengthening and stacking and then continues with widening and further stacking rearrangements.

By observing the changes in the crystallite size with slow cooling, we are also able to better understand how the mechanisms involved in the morphologies are observed in optical microscopy. In Figure 10b, we focus only on slow cooling because the specimens all had similar *X*_c_, whereas in fast cooling, *X*_c_ decreases with the addition of nanofillers, making it difficult to differentiate the change in crystallization from the decrease in *X*_c_.

Compared to Neat-Slow, the (110) crystallite size in CNC is higher, suggesting that CNCs either improve the extension of primary lamellae or initiate the formation of new ones. However, a lower (200) signal in CNC implies that lamellar widening and interlamellar infilling are reduced. We also observe that CNCs do not enhance the (111) stacking signal, and they reduce the complex stacking (211). Therefore, the radially aligned morphology has to be attributed to the CNCs’ improving lengthening while maximizing symmetric stacking interactions between lamellae.

In contrast, the CNC:GNP samples generally show a reduction in all crystallite size directions except for the stacking directions (111) and (211), suggesting that the highly branched morphology of CNC:GNP is achieved by minimizing the lengthening and widening mechanisms, while maximizing stacking. This suggests that vdW stabilization on graphite surfaces has a lower nucleating effect compared to Neat and CNC, but by minimizing nucleation, lamellar stacking interactions on the interface can be maximized.

In both nanofiller systems, we see that the crystallization mechanisms relative to Neat do not all improve, indicating that achieving direct increases in *X*_c_ in PEEK composites is not straightforward. Despite the degree of crystallinity decreasing in fast cooling or remaining the same in slow cooling, the fillers themselves directly alter the crystallization mechanisms in both positive and negative ways simultaneously, which directly influences the spherulitic morphology.

#### 3.3.2 Analysis of the XRD Spectral Shift

When the peak positions are compared relative to those of Neat, the dotted lines indicate that all samples with fillers induce right-shifting in the spectra, suggesting that the nanomaterials interact with PEEK by straining PEEK’s lattice. In Table 4, the spectral shift is converted to the changes in lattice spacing (*d*-spacing) of the PEEK primary diffraction planes. Higher Bragg angle (right shift) corresponds to smaller *d*-spacings. We observe that fast cooling results in smaller *d*-spacings due to fast cooling limiting the chain mobility, thereby leading to relaxation of the lattice. With slow cooling, right-shifting is still observed, suggesting that the shift here is correlated to the nucleation effect of the nanofillers and a mismatch in the thermal expansion of the fillers and PEEK. In both fast- and slow-cooled specimens, the *d*-spacing generally decreases with the addition of fillers, but for the fast-cooled CNC:GNP, we also observe a double peak, and broadening behavior is observed at 27°. The 27° signal present in the CNC:GNP samples corresponds to the GNPs (002) basal plane stacking (red). The peak splitting is likely attributed to the stress-induced reduction of graphite stacking symmetry during rapid cooling.^62^ CNC:GNP seems to exert a greater effect on lattice strain compared to CNCs, likely due the high thermal expansion mismatch between PEEK and GNPs because graphene is known to have a negative thermal expansion coefficient.

**Table 4:** XRD PEEK Crystallite Size (*L*) Perpendicular to the Plane (*hkl*) and Corresponding Interplanar Spacing (*d*-Spacing)

	(110)		(111)		(200)		(211)S	
specimen	*L* (nm)	*d* (nm)	*L* (nm)	*d* (nm)	*L* (nm)	*d* (nm)	*L* (nm)	*d* (nm)
Neat-Fast	12.75	0.467	12.83	0.424	10.25	0.387	9.42	0.308
CNC-Fast	12.32	0.459	12.71	0.417	10.08	0.381	9.38	0.304
CNC:GNP-Fast	11.45	0.455	12.73	0.413	9.50	0.378	9.16	0.303
Neat-Slow	14.16	0.469	14.55	0.424	11.48	0.387	11.33	0.308
CNC-Slow	14.30	0.459	14.58	0.417	11.07	0.381	10.98	0.304
CNC:GNP-Slow	13.73	0.461	15.02	0.418	11.18	0.382	11.32	0.305

However, why is the CNC signal not seen in the nanocomposites containing CNC? Aside from the possibility of peak merging due to lattice matching, the detection limit at a 1% concentration may not be sufficient for CNC capture, particularly if degradation effects further reduce the signal. While increasing the CNC concentration could enhance signal detection, this approach is not practical for manufacturing PEEK nanocomposites because degraded CNCs are unlikely to provide significant reinforcement. As a result, we aimed to verify via the literature that CNCs can partly maintain their crystal structure at elevated temperatures, which would support the ability of CNCs to in situ modify the crystallinity via lattice matching during PEEK nanocomposite manufacturing. A previous thermal decomposition study on the crystal structure of cellulose crystallites in wood revealed that the WAXS spectra of cellulose crystals experiences a left shift (increase in *d*-spacing) and a decrease of the signal intensity from 300 to 360 °C. This is indicative of lattice relaxation/expansion and degradation from decomposition.^63^ Given that our samples were manufactured under vacuum and under 15 MPa of compression, the oxidative degradation of cellulose was possibly minimized, as shown by the visible translucent CNCs in the microscopy images (Figure 6b). In the PEEK-CNC nanocomposites, we observe a reduction in the PEEK lattice spacing with CNCs, implying that the PEEK lattice is compressed with the inclusion CNCs. Because CNCs expand during degradation according to the aforementioned study, the compression effect on the PEEK lattice is logical. For GNPs, the lattice undergoes greater strain during fast cooling because the coefficient of thermal expansion of GNP is known to be significantly lower than that of PEEK. However, during slow cooling, and due to the lesser lattice match compared to CNCs, relaxation is more likely to occur. This implies that the internal residual strain of PEEK can vary according to the surface interactions with the nanomaterials. When changes in the *d*-spacing are combined with changes in the crystallite size, we can deduce that variations in the spherulite structure likely impose residual stresses on the PEEK lattice. In the case of CNCs, the straining effect appears to correlate with a relative increase in the lamellar lengthening (110) while maintaining symmetric stacking interactions (111). With GNPs, an enhanced lattice strain is observed with an increase in stacking. Therefore, considering the common influence on stacking, we hypothesize that the impact of lattice strain is primarily driven by how fillers predominantly affect lamellar stacking interactions.

#### 3.3.3 Proposed Nucleation Mechanism

The combined use of WAXS and polarized microscopy provides insights into how CNCs and GNPs affect the orientation and lamellar structure of spherulites at different cooling rates. However, a key question remains: What specific properties of CNCs contribute to the improved order and nucleation observed in optical microscopy? As previously discussed, studies suggest that GNPs induce edge-on nucleation according to vdW interactions, stabilizing PEEK chains in parallel to the graphitic basal planes. For CNCs, the answer may lie in the *a*-axis lattice parameter’s similarity to PEEK’s. In Figure 9a (blue lines), the spectra of pure CNCs films are shown. The *d*-spacing of the (200) plane for CNCs, calculated using Bragg’s law (*d*_CNC,(200)_ = 0.391 nm), suggests a monoclinic Cellulose Iβ structure based on the similarity of *d*-spacing derived from the published *a* lattice parameter of 0.7784 nm (*d*_CNC,(200)_ = 0.389 nm).^64^ Interestingly, *d*_CNC,(200)_ nearly coincides with the measured PEEK’s (200) (*d*_PEEK,(200)_ = 0.382–401 nm in Table 4). Unlike with GNPs, there is no distinct peak for CNC (200) in the PEEK-CNC nanocomposite spectra. This close lattice matching might be a key factor in inducing ordered PEEK spherulites. This could explain the lack of lattice relaxation observed with CNC in fast versus slowcooling. Because GNPs only stabilize the PEEK crystals according to vdW, the *a* lattice parameter similarities between CNC and PEEK may further constrain chain motion during cooling, explaining why the right shift is unchanging with CNC across cooling rates.

**Figure 11:**
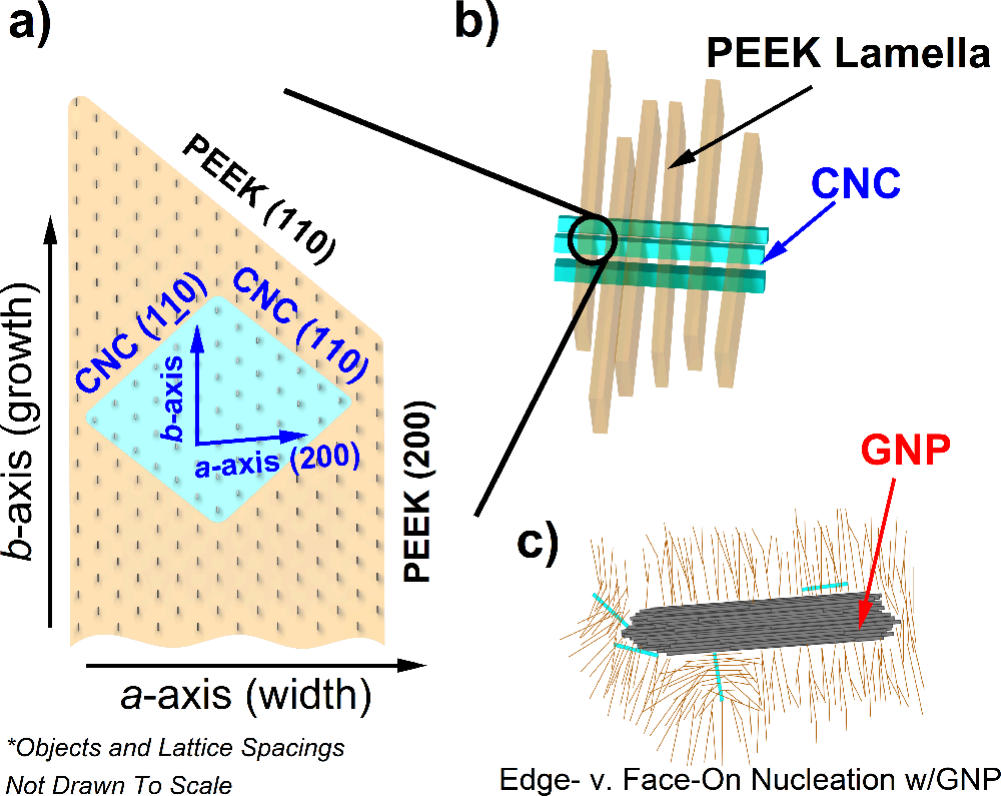
Schematic of the proposed CNC-mediated nucleation mechanism within PEEK. (a) Top-down view of CNC miscible within the PEEK lamella according to the expected (200) lattice match. (b) Isometric view of CNCs initiating nucleation via perpendicular pinning or growth of parallel lamellae. (c) Modification of PEEK nucleation on GNPs according to exposed surfaces without CNCs.

If lattice matching nucleation is plausible, CNCs might catalyze PEEK’s crystalline growth according to the mechanism in Figure 11. The matching of the (200) plane may facilitate direct miscibility of CNC within the PEEK lamellae. To optimize (200) lattice matching, CNCs’ *c* axis should be oriented to match the *c* axis of the PEEK unit cell, corresponding to a perpendicular orientation relative to the lamellae (Figure 11a). This orientation would support the radially symmetric structure from microscopy because a single CNC can pin several lamellae perpendicularly along its length, thus improving lamellar alignment while keeping the lengthening direction (110) unconstrained (Figure 11b).

In CNC:GNP-Slow, the lattice does not fully relax to match that of Neat, likely attributed to the trace amounts of CNCs affecting GNP surface nucleation. Figure 9 illustrates how CNCs may mediate the PEEK-GNP transcrystalline region. CNCs can either lay flat to maximize vdW interactions through the −OH hidden crystallographic planes or stand upright when covalently bonded to defect/edge sites in carbon nanomaterials.^57^ Due to the lack of lattice order on the GNP edges, PEEK is expected to nucleate face-on in these areas, but this could be affected by CNCs attached flat on the edges via vdW interactions. Similarly, CNCs may disrupt the edge-on nucleation on the basal planes if CNCs are oriented perpendicular via bonding to a vacant site. The conflicting nucleation processes may be the reason attributed to the highly branched nature of the CNC:GNP-Fast spherulites (Figure 5e). The next step is to determine how these microstructural changes influence the bulk degree of crystallinity (*X*_c_) and the resulting macroscale mechanical properties of the nanocomposite.

#### 3.3.4 SAXS

**Figure 12:**
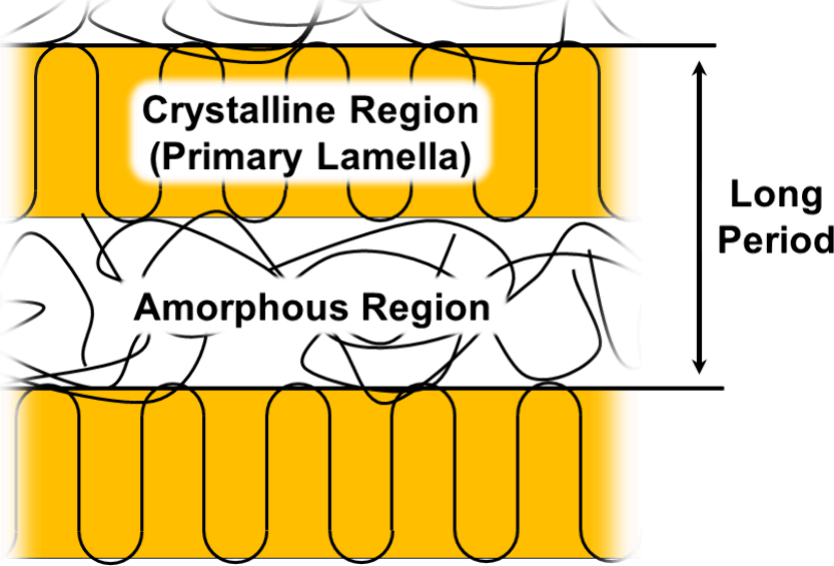
Schematic representation of the long period, the average periodic distance between two lamellae that accounts for both crystalline and amorphous regions.

To quantify the lamellar structure of the spherulites formed during the cooling process, the semicrystalline peaks were analyzed to calculate the long periods of each sample. The long period represents the average periodic thickness of the lamella and the amorphous regions along crystalline PEEK’s [001] direction (Figure 12).

**Figure 13:**
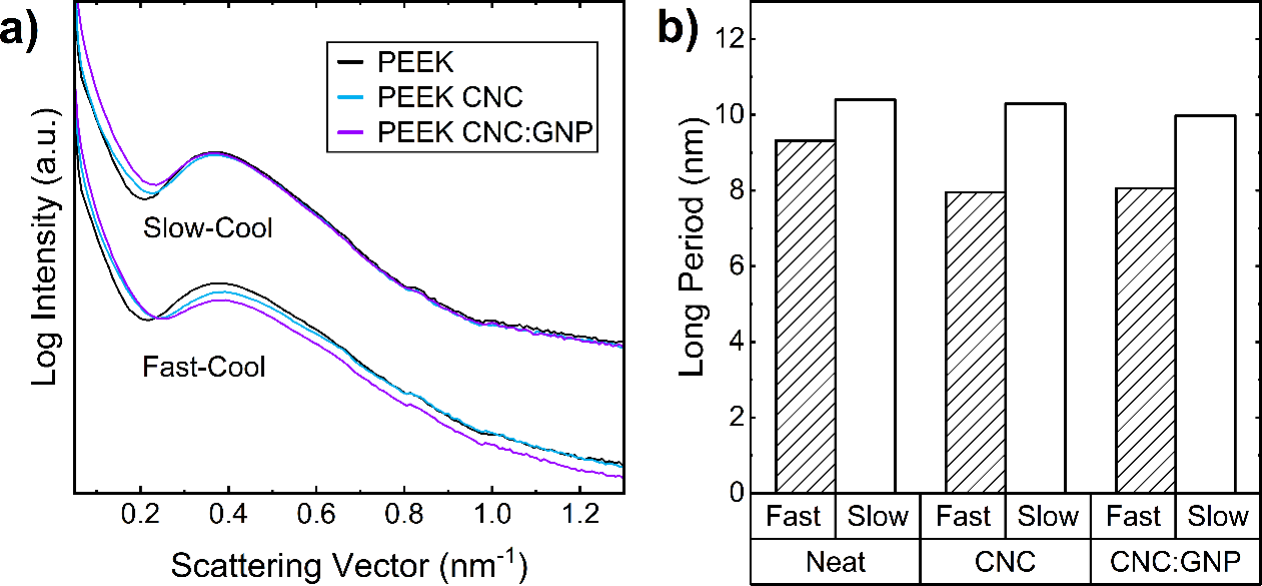
(a) Raw SAXS spectra of PEEK nanocomposites, indicating a semicrystalline peak at *Q* = 0.37 nm^–1^. (b) Calculated long period for each of the PEEK nanocomposites using the semicrystalline peak in the SAXS spectra.

The SAXS patterns shown in Figure 13a demonstrate a semicrystalline peak across all six nanocomposite concentrations and cooling rates, signifying the presence of crystalline domains within all of the samples. As shown in Figure 13b, the long period of nanocomposite samples decreases as a result of both the fast-cooling rate and the addition of nanoparticles to the PEEK matrix. Samples that were fast-cooled produced smaller long periods than their slow-cooled counterparts because of the limited chain mobility during the rapid temperature decline, which prevented the growth of crystallites within the matrix. The addition of CNC and CNC:GNP generally leads to a decrease in the long period, suggesting a less dense crystalline structure at first glance. DSC measurements of the bulk crystallinity (proceeding Section 3.3) tell a similar story, showing a decrease in *X*_c_ with fast cooling or no change with slow cooling (Figure 8b). However, if analysis of the crystallite size in Figure 10 suggests changes in stacking interactions, then a reduction in the crystallite size might not solely indicate a decrease in *X*_c_ but could also imply an enhancement in stacking interactions, leading to a shorter long period. Although DSC results show no change or an increase in *X*_c_ when CNC and CNC:GNP are added and subjected to slow cooling, we observe a slight reduction in the long period under the same conditions with both fillers. This discrepancy suggests that changes in stacking interactions may significantly affect the structural dynamics within the composite, which are not fully captured by *X*_c_ alone.

Although the long period data seemingly do not match the crystallinity data from DSC, it is imperative to note that DSC evaluates the bulk material, capturing its overall behavior without being affected by the orientation of the crystallites, unlike X-ray scattering and microscopy, which are respectively influenced by the measurement geometry (discussed with Figure S10) or the sample preparation (thin films required for transmission optical microscopy). The material’s crystallites are generally randomly oriented, yet WAXS and azimuthal angle plots (discussed alongside Figures S10 and S11) have confirmed a preferential alignment parallel to the surface due to resin flow. Due to alignment effects with the beam, there are crystallite structures that are perpendicular to the incident beam direction, which are not detected in this data set. However, XRD analysis does confirm that CNCs and CNC:GNPs contribute to the creation of a more populus, and possibly thinner, lamellar network that supports the findings from DSC characterization (Section 3.1) and the suggested nucleation mechanism in Figure 11.

### 3.4 Mechanical Properties of PEEK Nanocomposites

**Figure 14:**
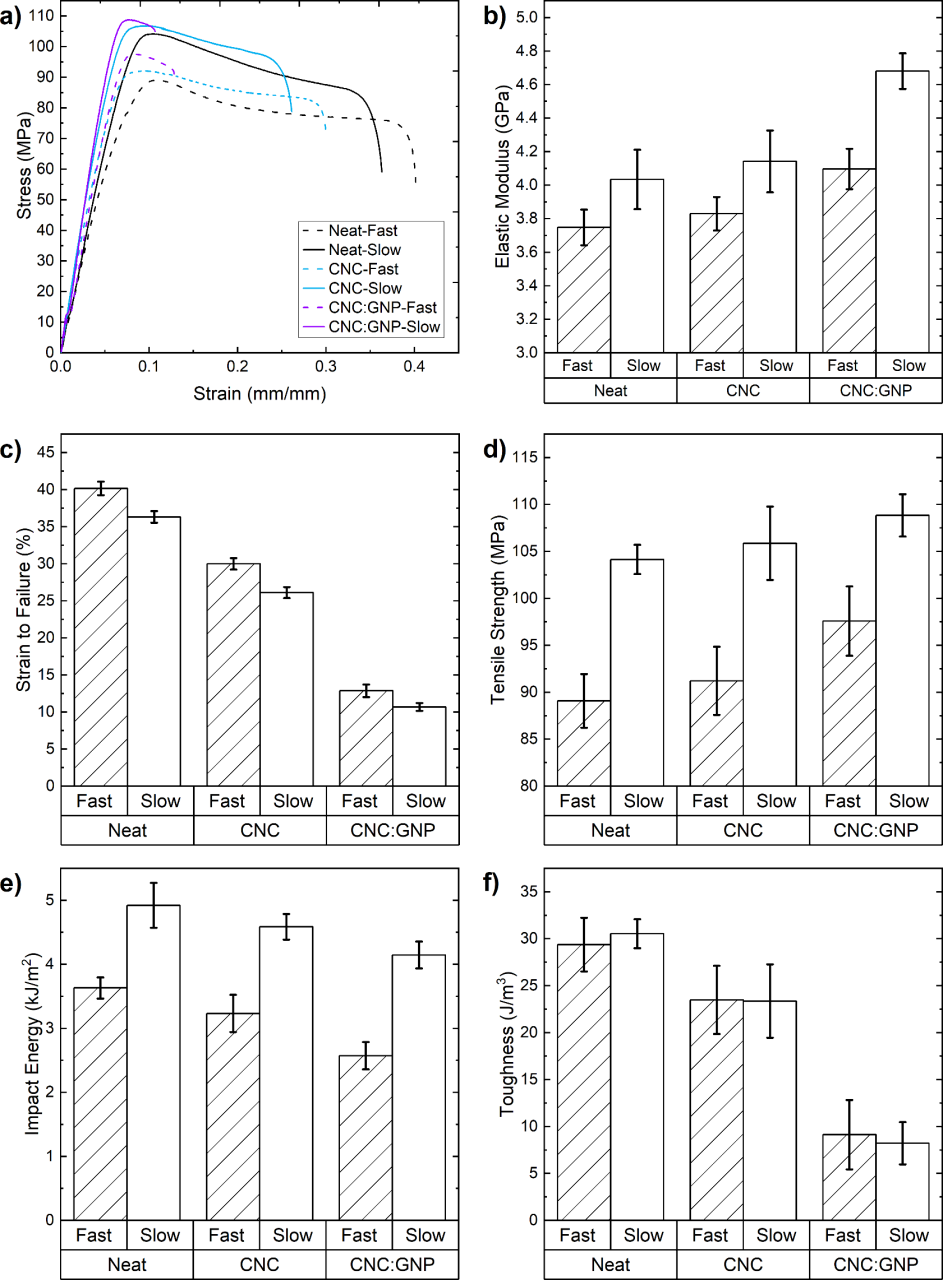
(a) Sample engineering tensile stress–strain curves of the PEEK nanocomposite specimens. (b–f) Comparison of the mechanical properties derived from tensile and Izod impact tests.

Because crystallization is known to influence properties related to both low- and high-strain-rate crack propagation, tensile tests and Izod impact tests allow us to analyze how changes in the crystalline microstructure affect the mechanical behavior of the nanocomposites. Figure 14a summarizes the tensile behavior of the nanocomposites, highlighting that the mechanical performance is heavily influenced by changes in the crystallinity and crystalline morphology, which is dependent on the composition and cooling rate. In Figures 8b and 14, we observe the overarching effect of crystallinity. The spherulite size and morphology influence the crack propagation resistance and the overall mechanical behavior at both low and high strain rates. This is expected because the mechanical response of a polymer is dependent on the chain mobility; densely packed chains in the crystalline regions typically result in a higher stiffness, leading to brittle fracture behavior at higher degrees of crystallinity. Conversely, fast cooling, which yields a higher amorphous content, results in a lower stiffness with greater ductility.

Although CNCs alter the crystallinity and spherulite morphology, the strength and elastic modulus was only improved by 2–3% in both fast and slow cooling. In contrast, the CNC:GNP system resulted in a ∼9.5% increase in the strength and modulus for fast cooling and 4.5% and 16% increases for the strength and modulus in slow cooling. The stark difference in improvement between CNC and CNC:GNP suggests that CNC alone provides very limited mechanical reinforcement compared to GNPs, especially when the effect of thermal degradation is included. CNCs are expected to reduce the mechanical response, especially as the crystallinity is reduced in fast cooling (Figure 14b), yet here the combination of CNC:GNP is shown to improve the mechanical behavior by improving the quantity and altering the crystalline morphology and architecture during solidification. Therefore, we find that CNCs alone cannot be used to reinforce PEEK because they must be paired with a secondary filler to produce a unique 3D spatial geometry to nucleate and build a tailored crystalline architecture that can provide the desired mechanical and functional properties. The morphology of the primary filler takes precedence in governing the mechanical properties, but CNCs can be used to tune the crystal structure in the interphase region. We note that CNC:GNP decreases the strain of failure by 68% despite improving all other metrics. This decrease in the work of fracture at low strain rates may be a consequence of low interface adhesion from noncovalent dispersion and a higher degree of crystallinity and change in the spherulite morphology. Additionally, the inclusion of larger bonded nanoparticles, specifically CNC:GNP, constrains the chain motion within the material, resulting in a brittle fracture mechanism exhibited by the lower strain at break values.

Another consideration is the spherulite size and uniformity, as influenced by CNC and CNC:GNP. Under fast-cooling conditions, CNC predominantly results in small, densely packed spherulites, while slow cooling leads to significant variations in the crystal structure (Figure 5c,d): the CNC-Slow specimens contained significant regions of randomly infilled dendrites (Figure 5d), and the spherulites are mainly concentrated around CNCs (Figure 6). In contrast, the Neat samples contained slightly larger spherulites with moderate branching, whereas the CNC:GNP samples feature the largest branched spherulites in fast cooling but spherulite sizes similar to those of the Neat samples in slow cooling (Figure 5a,b,e,f). Interestingly, despite the reduction in crystallinity for CNC-Fast, it still notably improves the strength and modulus, suggesting that modifying the crystalline structure plays a more critical role than crystallinity in influencing the mechanical response in fast-cooling rates. Despite these microstructural variations, the measured toughness decreases with the introduction of fillers but remains roughly equal across both fast and slow cooling for each composition, implying that plastic flow during quasi-static loading is dominated by the composition. The decrease in toughness with CNC and CNC:GNPs might be due to the decreased chain mobility with CNC and size effects generating local stress concentrations with GNPs.

Parts e and f of Figure 14 compare the tensile toughness and Izod impact response of the nanocomposites, providing a comparative analysis of the crack propagation resistance at low and high strain rates. As previously mentioned, composition is the factor that dictates energy absorption at low strain rates because the toughness remains the same with varying cooling rates with quasi-static loading conditions. At high strain rates, the situation is similar but potentially more nuanced. Factors such as the crystallinity, spherulite size, morphology, and filler loading size can all directly contribute to changes in crack propagation resistance. Yet, our findings show that slower cooling generally improves impact resistance, while the addition of CNC and CNC:GNP tends to reduce it (Figure 14f). This indicates a direct relationship between the energy absorption capabilities, crystallinity, and inclusion of nanomaterials.

Perkins’ review on the structure–property relationships in polymers indicates that the spherulitic morphology can substantially influence the mechanical response, whereas smaller spherulite sizes typically lead to higher plastic flow and higher impact strengths. He notes that trends in deformation and crack propagation in semicrystalline polymers often occur through the edges of the spherulites along the amorphous regions, where large spherulites tend to offer less resistance to crack propagation,^65^ likely due to the limited ability to deflect cracks. Establishing a direct correlation between the impact and spherulite morphology in our study is challenging because we observe differences in the spherulite size with the same cooling rates. Despite CNC samples showing the finest structure from optical microscopy, the toughness and impact resistance are lower than those of the Neat samples, indicating that the overall crystallinity plays a stronger role in the mechanical response than spherulite morphology alone.

The influence of crystallinity on the impact resistance at high strain rates seems counterintuitive at first because a greater amorphous content should allow for improved energy dissipation via plastic deformation. Without any clearly defined crystalline structure, cracks can freely propagate, thereby requiring some degree of crystallinity to improve crack deflection. This theory supports why slow cooling generally improves the impact resistance across all compositions. This is corroborated by a previous study on the Izod impact resistance of injection-molded PEEK,^7^ where a lower degree of undercooling in injection-molded PEEK resulted in higher energy absorption, reinforcing the idea that crystallinity can improve the impact resistance under drastic variations in the cooling rate.

However, the inclusion of nanomaterials adversely affects the impact performance because they likely serve as crack initiation points within the material. Similar results in the filler/matrix compatibility of clay/polypropylene–polyethylene nanocomposites have pointed to the same trend where the impact resistance improved with polypropylene but decreased with polyethylene despite similar levels of crystallinity.^66^ In our case, the strong nucleation effect of CNCs is undermined by its thermal degradation, thereby providing a reduction on the impact energy (11% for fast cooling and 7% for slow cooling). The larger GNPs with comparatively limited influence on nucleation in addition to larger size significantly reduce the impact energy (39% for fast cooling and 16% for slow cooling). The more pronounced reduction with fast-cooled CNC:GNP suggests that in this study nanomaterials themselves do not inhibit crack propagation; rather, they facilitate it. While CNC:GNP provides the greatest improvement in stiffness and strength, careful consideration of the nanomaterial selection and processing conditions is required to maximize the mechanical performance for specific applications.

## 4 Conclusion

In this study, we introduced a scalable and facile fabrication methodology for PEEK nanocomposites, employing a hybrid nanomaterial system, namely, CNC-bonded GNP with a unique spatial geometry, to modify the crystalline architecture. Typically, it is observed throughout the literature that fillers accelerate the crystallization rate of PEEK, but the overall degree of crystallinity does not significantly change. Here our study highlights that readers should not necessarily focus on just the degree of crystallinity itself but the influence of the fillers on the structure of the crystals because the structure may also play a key role in dictating the mechanical response of the nanocomposite. The use of select fillers that modify the crystal morphology is shown to mitigate the effects of low degrees of crystallinity typically found in PEEK-GNP nanocomposites, particularly during fast cooling. The work presented in this study demonstrates a solution for polymer manufacturers that is easily implemented in existing systems with rapid-cooling environments, such as injection molding and fused filament fabrication. By using water with CNCs as a dispersion medium for the nanomaterials, there are no additional hazards or wastes that manufacturers will be required to consider, allowing for high-volume manufacturing processes to overcome current obstacles with low crystallinity due to fast cooling. Despite processing the panels under vacuum, thermal degradation of CNC is a key factor that cannot be avoided due to the lower thermal stability of single-bonded oxygen species present in CNC. However, our findings highlight that nonbonding interactions and the crystal structure compatibility between fillers and PEEK are the primary factors influencing the morphological changes of the PEEK spherulites. Therefore, we propose that the selection of a thermally stable, inorganic filler that exhibits this compatibility is the key to maximizing the mechanical performance of PEEK composites.

Through polarized optical microscopy and XRD analyses, we observed significant microstructural changes in the crystalline morphology, providing insights into how the cooling rate and nanomaterial morphology can be cotailored to influence the spherulite growth mechanisms. The crystalline morphology achieved by the introduction of CNC:GNP to the PEEK matrix simultaneously translates into a higher mechanical performance through increased tensile strength by 5% and tensile modulus by 16%. However, the combined use of the cooling rate with a hybrid nanomaterial system requires a careful balance of trade-offs to maximize the mechanical performance and manufacturing scalability. Our work exemplifies that the modification of PEEK’s microstructure is achievable by tailoring the crystalline morphology, thus improving the mechanical performance of PEEK in fast-rate manufacturing processes. However, future work is needed to verify the direct correlation between the spherulite size and morphology on the composite’s behavior because the fillers’ inclusion significantly affects the composite’s mechanical behavior.

## Supporting Information Available

Images and detailed descriptions of the powder preparation process, molding and annealing operations, image of a PEEK-GNP nanocomposite spherulite, tabulated WAXS peak parameters, comparison of the XRD and WAXS spectra, SAXS azimuthal intensity plot and analysis, and modulated DSC scans of amorphous PEEK (PDF)
