# Supplementary material for: Engineering the Crystalline Architecture for Enhanced Properties in Fast-Rate Processing of Poly(ether ether ketone) (PEEK) Nanocomposites
Source: ACS Appl Eng Mater. 2024 Aug 7;2(8):2038–54. doi: 10.1021/acsaenm.4c00217 (PMC11348426; doi:10.1021/acsaenm.4c00217)
Supplement: Supplementary file 1 — em4c00217_si_001.pdf [file em4c00217_si_001.pdf]

Supporting Information: Engineering the Crystalline Architecture for Enhanced Properties in Fast Rate Processing of Polyether Ether Ketone (PEEK) Nanocomposites

Behrooz Shirani Bidabadi[1§], Emile Motta de Castro[2§], Mia Carrola[3], Pratik Koirala[4], Mehran Tehrani[4 5 6] and Amir Asadi,[1,3]*

[1] Department of Engineering Technology and Industrial Distribution, Texas A&M Universitveryy, College Station, Texas 77843-3367, United States

[2] J. Mike Walker '66 Department of Mechanical Engineering, Texas A&M University, College Station, Texas 77843, United States

[3] Department of Materials Science and Engineering, Texas A&M University, College Station, Texas 77843, United States

[4] Walker Department of Mechanical Engineering, University of Texas at Austin, Austin, Texas 78712-1591

[5] Department of Structural Engineering, University of California San Diego, La Jolla, CA, USA

[6] Program in Materials Science and Engineering, University of California San Diego, La Jolla, CA, USA

[§]Behrooz Shirani Bidabadi and Emile Motta de Castro contributed equally to this work

**Corresponding Author,* Amir Asadi: amir.asadi@tamu.edu

S1 ADDITIONAL INFORMATION ON THE MANUFACTURING PROCESS

S1.1 Preparation of the Powder Charge

The schematic view of the powder preparation process is shown in **Figure S1**. For the CNC-PEEK panel, CNCs were dispersed in distilled water using ultrasonication for an hour in an ice bath as described in the manuscript.

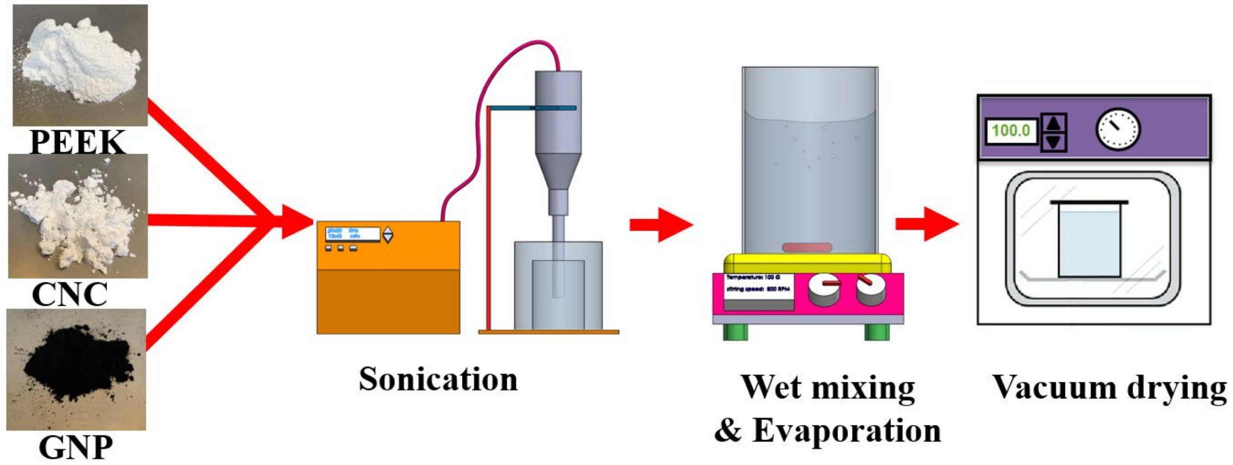

Figure S1. Schematic view of Powder preparation process.

Then PEEK powders were added to the suspension until no PEEK powders remained floating. Then the suspension was placed on a hot plate and magnetically stirred until the majority of the water was fully evaporated. Finally, the vacuum oven was used for 24 hours at 100^{0}C under the vacuum to fully powder. **Figure S2** shows the suspension when the PEEK powders were added and during evaporation.

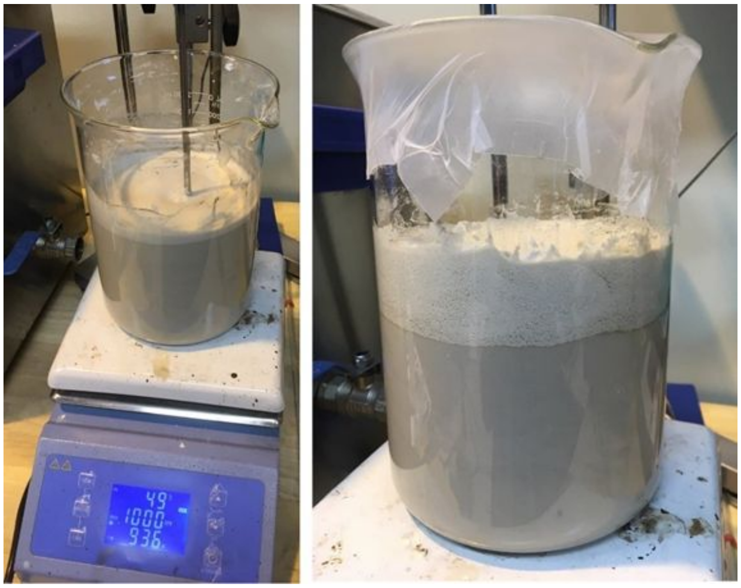

Figure S2 PEEK-nanoparticle aqueous suspension steering on the hot plate

The steps in the PEEK-CNC/GNP composite panel are the same as in the PEEK-CNC panel, except for the procedure needed to add GNP into the suspension. GNP is not dispersed very well in distilled water; most of the time, it agglomerates if we don't use CNC nanoparticles. So, CNC was used as a dispersive particle in the distilled water for any composition requiring the GNP to be mixed with PEEK. **Figure S3** compares the dispersion stability of GNP and CNC:GNP in water. The same steps were performed with the PEEK-CNC powders, except GNPs was added to the solution after 1 hour and sonicated for 1 additional hour.

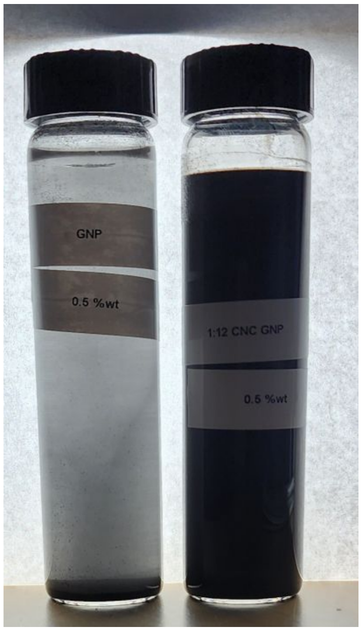

Figure S3. Vial images indicating dispersion and stability of GNP and CNC:GNP 1:12 in water 0.5% wt.

S1.2 Molding Process

The hydraulic press produced adequate pressure for void-free moldings, reaching 350 bar pressure inside the mold cavity during the packing stage. This was possible because of the press's ability to deliver sufficient force up to 10 kN. The hydraulic press was outfitted with two heated platens, each having the capacity to heat the mold to temperatures of up to 400 °C, making it suitable for PEEK-based composites.

A mold release agent was utilized to complete the ejection process more quickly. In order to accomplish this goal, Loctite Frekote 44-NC Release Agent was used. This agent was designed to be stable at temperatures higher than the highest temperature attained throughout the production cycle. Six separate layers of mold release agent were brushed over all the surfaces of the mold in order to ensure that an adequate coating of the substance was present. To complete the molding process, the following procedures were carried out:

All components were meticulously cleaned using isopropyl alcohol to prevent contamination before assembly, as shown in **Figure S4**.

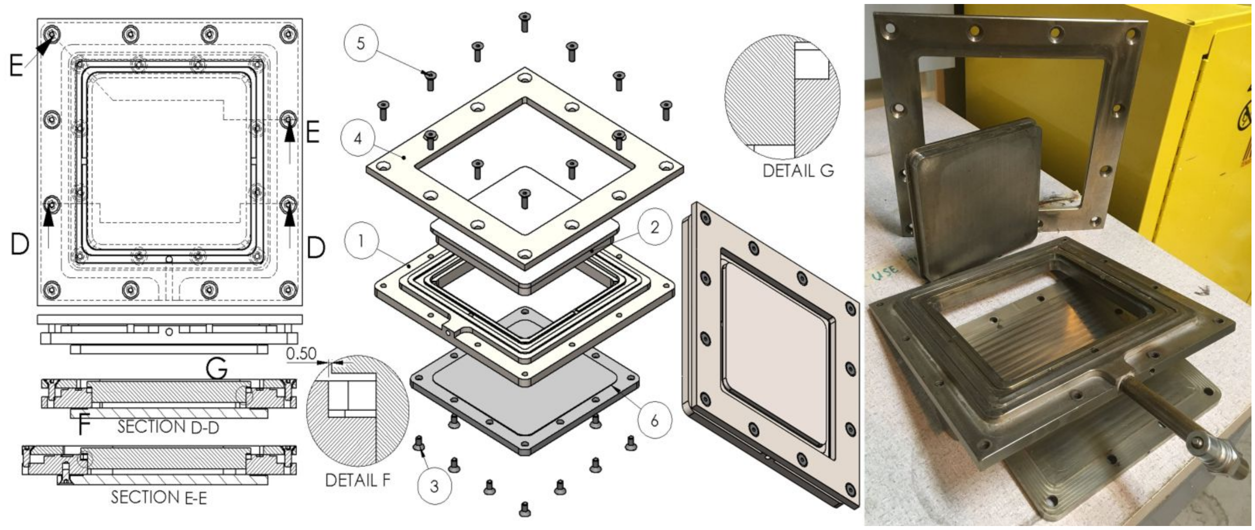

Figure S4. Compression mold assembly before the molding process

Composite powders were dried before being molded; the drying process for the powders took three hours at a temperature of 110 degrees Celsius. The necessary amount of powder needed to create the panels could be approximately determined by using **Equation S1**:

$$m_p = V\rho \cdot \text{(S1)}$$

where m_p is the mass of the powder charge, V is the desired volume of the panel, ρ is the density of the powder charge using rule of mixtures, and 1.02 is a scaling factor to account for loss from resin squeeze-out.

The piston was positioned on top of the cavity. Then sealant tape was placed in the cavity margin, and a vacuum bag covered the whole mold, including the piston as shown in **Figure S5**. Finally, the vacuum frame was bolted down to the cylinder.

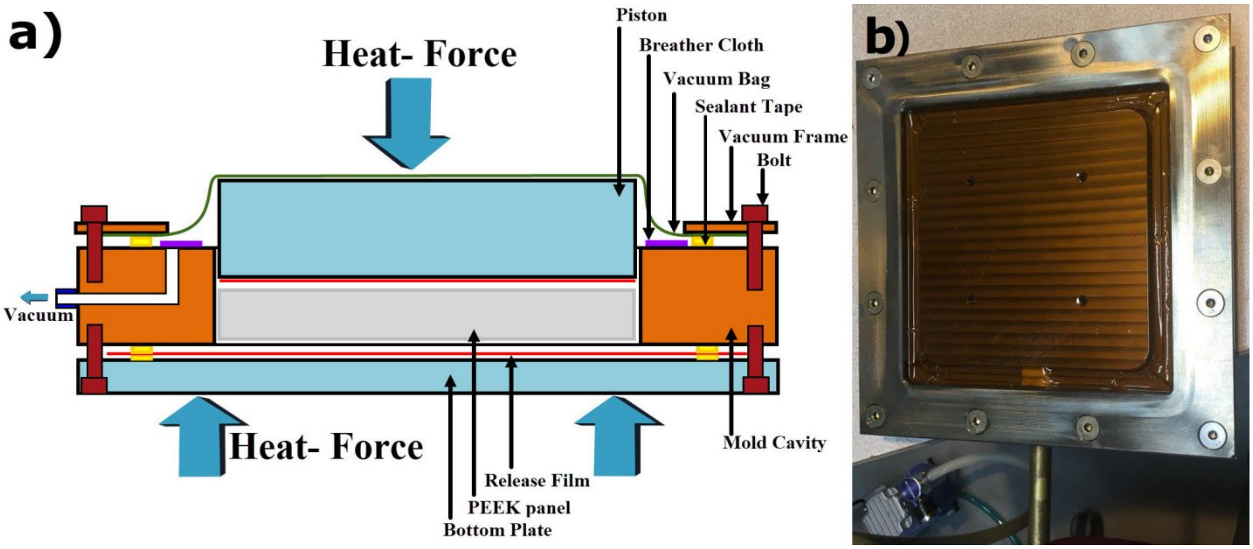

Figure S5. schematic view of molding process and Vacuum sealed mold before going under the heating and compression process

The hydraulic press was used to provide high pressure of 350 bars inside the cavity to pack the powder while it was at room temperature under the vacuum. The heaters for the platens were preheated to 380 degrees Celsius and quickly heated the mold while maintaining a cavity pressure of fewer than 20 bars. Once the system achieved the appropriate temperature, the mold was kept at constant temperature and pressure for 20 minutes to create homogeneity within the melt. The Platens' heating was turned off, the pressure was raised to 350 bar, and the system was allowed to reach room temperature before being shut off. For ejection of the composite panel, the mold was opened from the back in order to remove the composite panels (**Figure S6**).

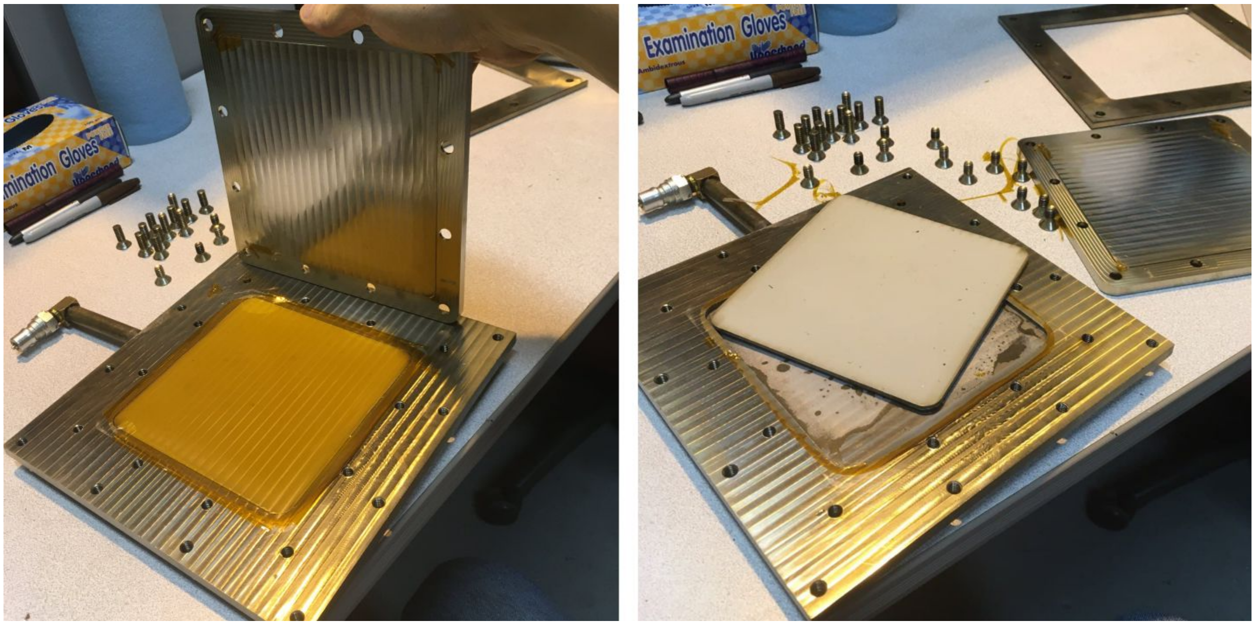

Figure S6. Compression mold opens up after the molding process and taking out the PEEK panel

S1.3 Annealing process

The annealing process was used for the samples with a fast-cooling rate. The same procedure as mentioned for the molding process was followed in the annealing process, except that the materials were composite panels, not composite powders. So, the composite powders were molded at the first step and turned into composite panels using compression mold. In the second step, the panels were annealed using the annealing mold. **Figure S7** shows the annealing steps.

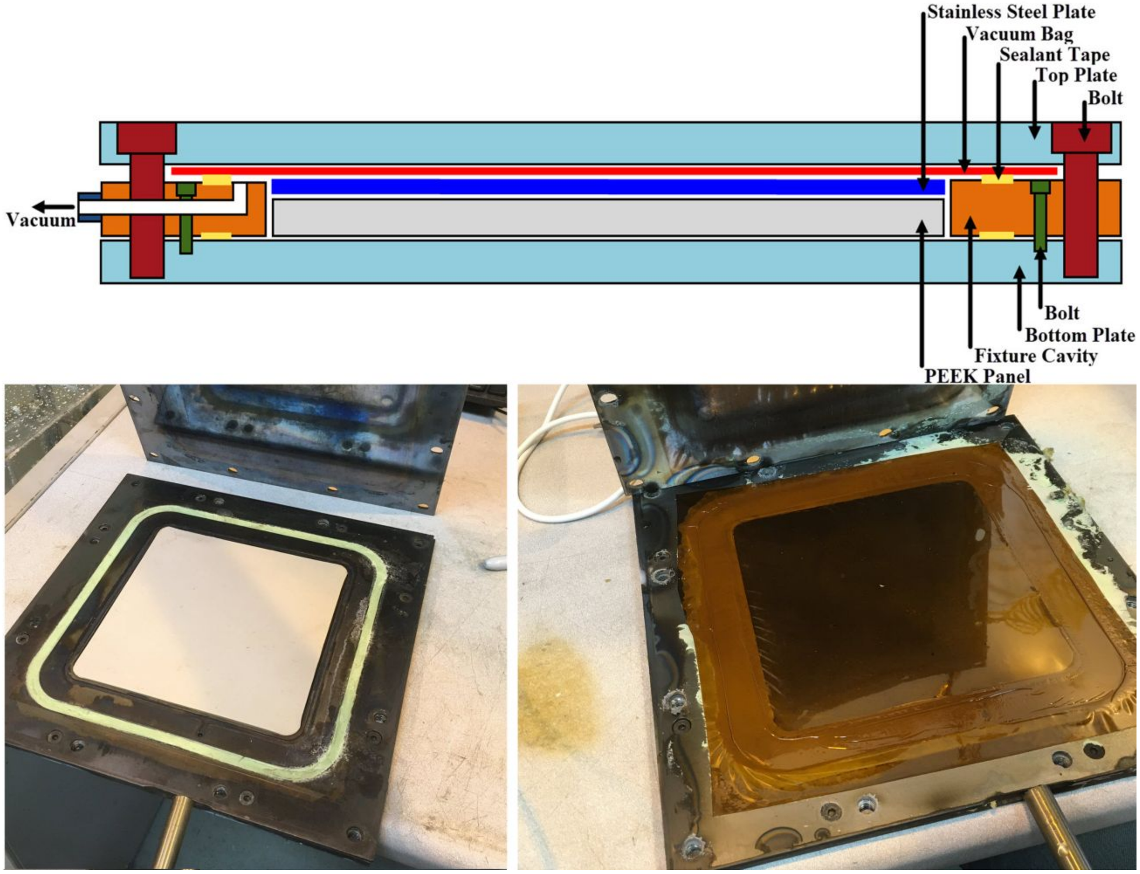

Figure S7. Annealing mold process includes part assembly, location PEEK panel, and vacuum sealing

In the annealing process, first the annealing mold parts were cleaned and coated with the mold release agent. Then the cavity frame was bolted down to the back plate and sealant tape was used in the margin of the cavity as a gasket. The composite panel was placed in the mold cavity as seen in **Figure S7.** Then a vacuum bag covered the panel, and the top plate was bolted down to the bottom. The hydraulic press was turned on, and the platens were heated up to 375^0C. The mold was placed in the hydraulic press, and the pressure increased to 350 bar and kept for 15 minutes to make sure the panel fully melted. The pressure was released, and the annealing fixture was removed from the hydraulic press at 375^0C. Finally, the annealing mold was cooled to room temperature at different cooling rates. Depending on the required rate of cooling, a variety of different protocols could be carried out, such as a rapid cooling process using high pressure water bath as shown in **Figure S8** or a slow cooling process in which the platens and mold are allowed to cool down in the ambient temperature, all while maintaining vacuum in the mold.

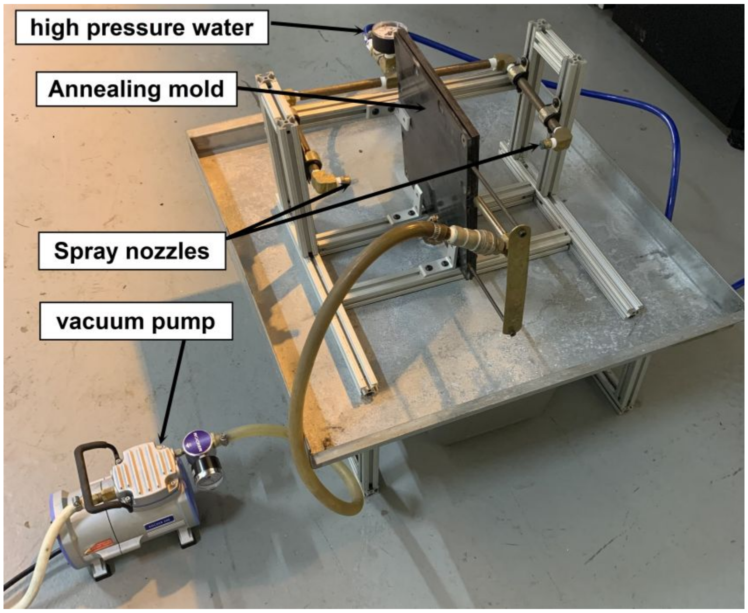

Figure S8. High pressure water bath for fast cooling of the annealing mold.

S2. SUPPLEMENTARY RESULTS

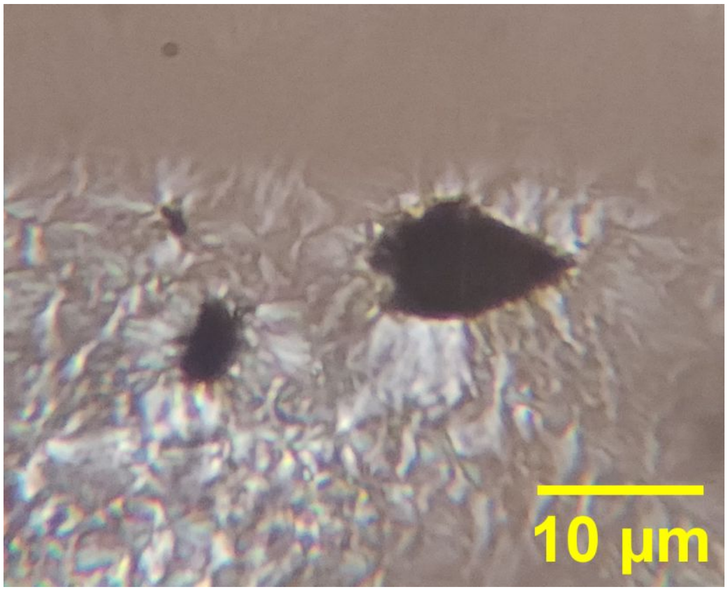

Figure S9. Polarized optical microscopy of PEEK with pristine GNPs, indicating edge-on nucleation of PEEK on GNP surfaces.

Table S1. WAXS spectra peak positions and calculated full width half-max (FWHM) of peaks

Specimen	(110) 2θ (°)	(110) FWHM (°)	(111) 2θ (°)	(111) FWHM (°)	(200) 2θ (°)	(200) FWHM (°)	(211) 2θ (°)	(211) FWHM (°)
Neat-Fast	19.25	0.702	21.25	0.700	23.26	0.879	29.34	0.967
CNC-Fast	19.03	0.727	21.03	0.707	23.03	0.894	29.11	0.973
CNC:GNP-Fast	19.25	0.783	21.25	0.706	23.26	0.949	29.34	0.997
Neat-Slow	18.36	0.632	20.14	0.617	22.15	0.785	28.21	0.805
CNC-Slow	19.03	0.626	20.81	0.616	22.82	0.814	28.88	0.831
CNC:GNP-Slow	18.81	0.652	20.81	0.598	22.59	0.806	28.88	0.806

To complement the XRD findings, WAXS confirmed preferential alignment effects within the specimens. WAXS was performed using the same system as SAXS from the main manuscript (Xenocs Seuss 3.0). **Figure S10** compares the spectra across cooling rate and composition, showcasing variations in peak broadening, shifting, intensity, and the presence of the GNP signal. Unlike XRD, which uses reflection geometry focusing on lattice planes parallel to the surface, WAXS employs transmission geometry, highlighting planes perpendicular to the surface. This difference in geometry likely explains the more prominent detection of PEEK (200) in XRD, and a higher detection of PEEK (110) in WAXS. The peak shift and broadening effects observed in WAXS can likely be attributed to further scattering/absorption effects as the 3mm thickness of the bulk specimen is atypical for transmission measurements. Interestingly, we find that the (002) GNP signal is muted in WAXS, likely due to lack of alignment and scattering of the signal with transmission geometry. These results reveal that the compression molding methodology introduces preferential alignment of the crystalline structure parallel to the surface, with WAXS detecting more (110) due to spherulitic growth primarily forming primary lamellae without significant lamellar thickness increases across the thickness of the panel. This growth pattern comparing WAXS and XRD, restricted by the sample's thickness, suggests that lamella widening and secondary crystallization mainly occur parallel to the surface, resulting in a stronger (200) signal in XRD's reflection geometry. The absence of GNP detection in WAXS indicates GNPs align parallel, not perpendicular to the surface, likely due to flow-induced alignment during compression molding. **Figure S11**'s SAXS azimuthal intensity plots further demonstrate alignment effects, with the sinusoidal signal for CNC:GNP-slow samples suggesting slight unidirectional alignment due to resin flow during molding, as the samples were cut offset from the center of the panel.

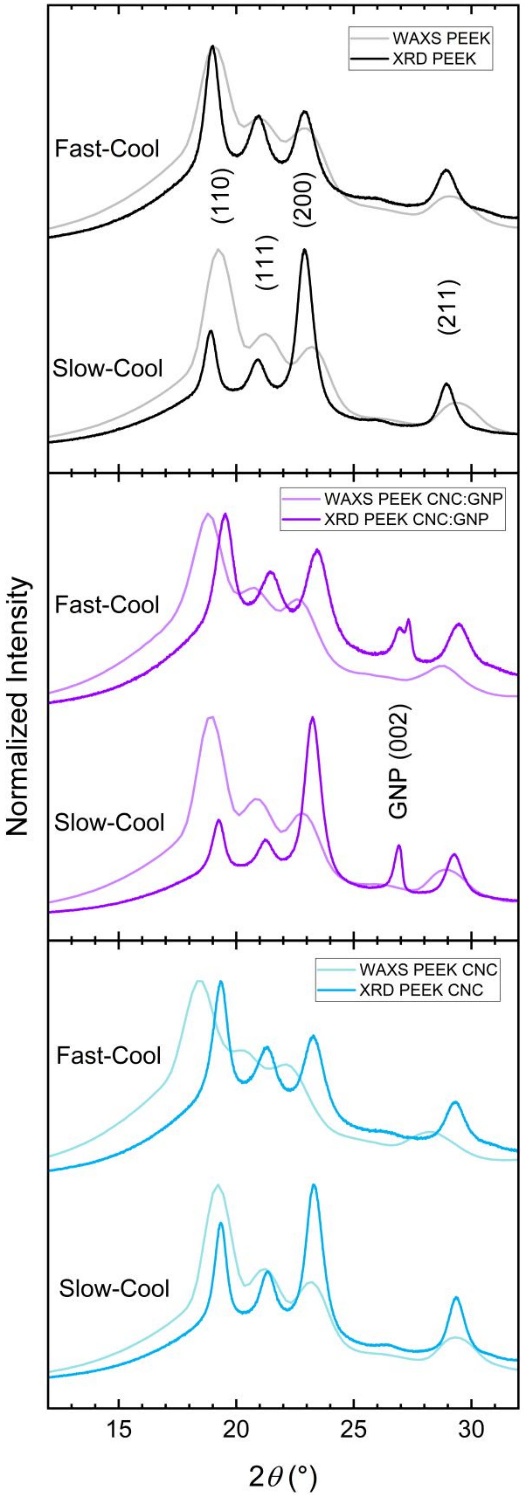

Figure S10. Comparison of WAXS and XRD spectra showcasing how measurement geometry (reflection v. transmission) influences the observed spectra.

To assess any potential alignment effects caused by the introduction of the nanomaterials, we analyzed the azimuthal angle plot using the scattering vector value of $Q = 0.25$ nm^{-1} for any periodic signals.[1] A subtle alignment was detected only in CNC:GNP-Slow, indicated by the sinusoidal trendline in red (**Figure S11**). We hypothesize that the slow-cooled crystallites nucleated on the GNPs and oriented normal to the X-ray beam, resulted in the observed periodic signal. The same scattering pattern would likely occur if the sample were mounted differently during the scan, given the random orientation of GNPs within the sample. Ultimately, this finding emphasizes the influence of CNC:GNP on both nucleation and alignment directionality. However, as the material is rendered isotropic due to the manufacturing process, this alignment is not significant enough to introduce macroscopic anisotropy in this instance.

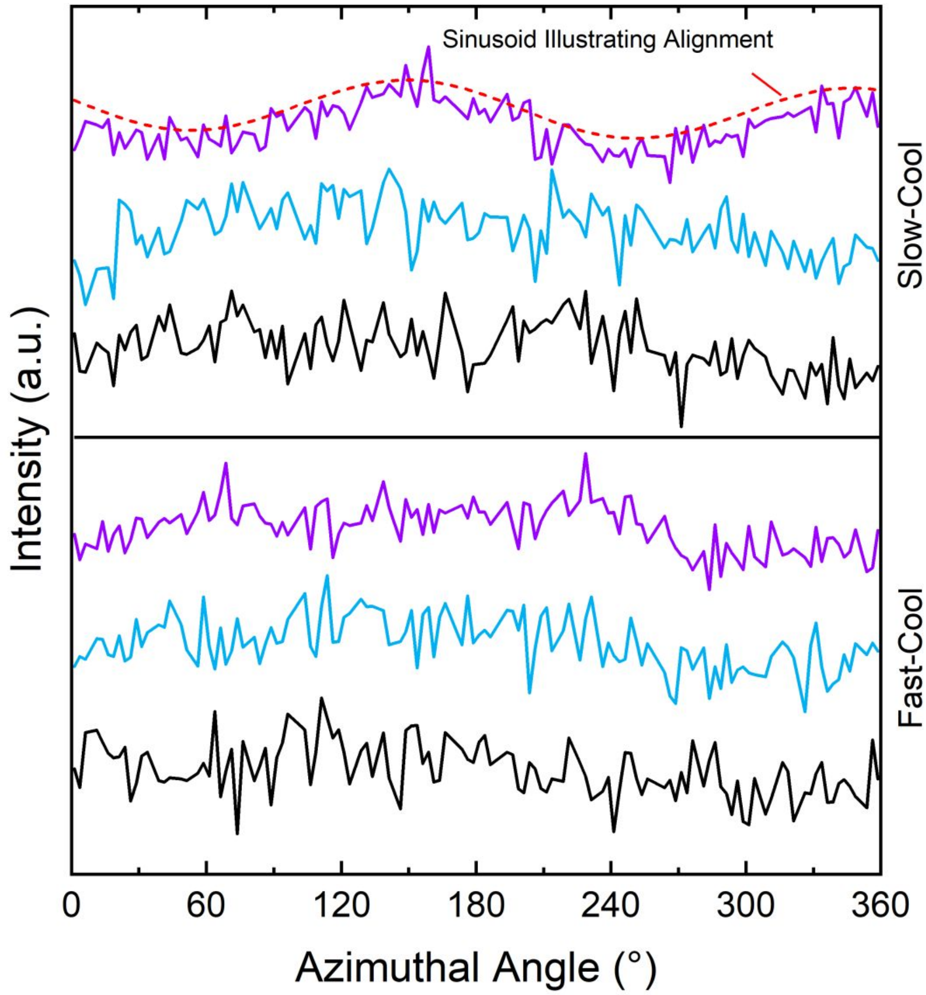

Figure S11. SAXS azimuthal intensity plot for $Q = 0.25$ nm^{-1}. Red sinusoidal trend on CNC:GNP-Slow signifies presence of alignment.

Figure S12 provides a modulated differential scanning calorimetry (MDSC) heating scan of amorphous PEEK to assist in identifying the cold crystallization enthalpy and T_g temperatures.

From **Figure 11** in the main manuscript, an exothermic signal near 150˚C appears, suggesting cold crystallization. Yet, the MDSC scan reveals that cold crystallization should appear as a separate peak, at least 15-20˚C higher than the location of the T_g for the grade of PEEK used. The discrepancy in the location of T_g and melting enthalpy from **Figure 11** stems from the differences in heating rate applied (1˚C v. 10˚C).[2] The absence of a cold crystallization peak in **Figure 11** suggests that despite the rapid cooling of the mold, the nanocomposite panel did not sufficiently cool at a high enough rate to produce cold crystallization during the DSC heating scan. The change in shape of the T_g signal from **Figure S12** and **Figure 11** can be attributed to the difference in amorphous content of the specimens, as well as possible stress relaxation. As T_g is dictated by a signal change attributed to chain relaxation and mobility, a higher crystallinity has shown to limit the stepwise T_g signal that is typically observed.[3]

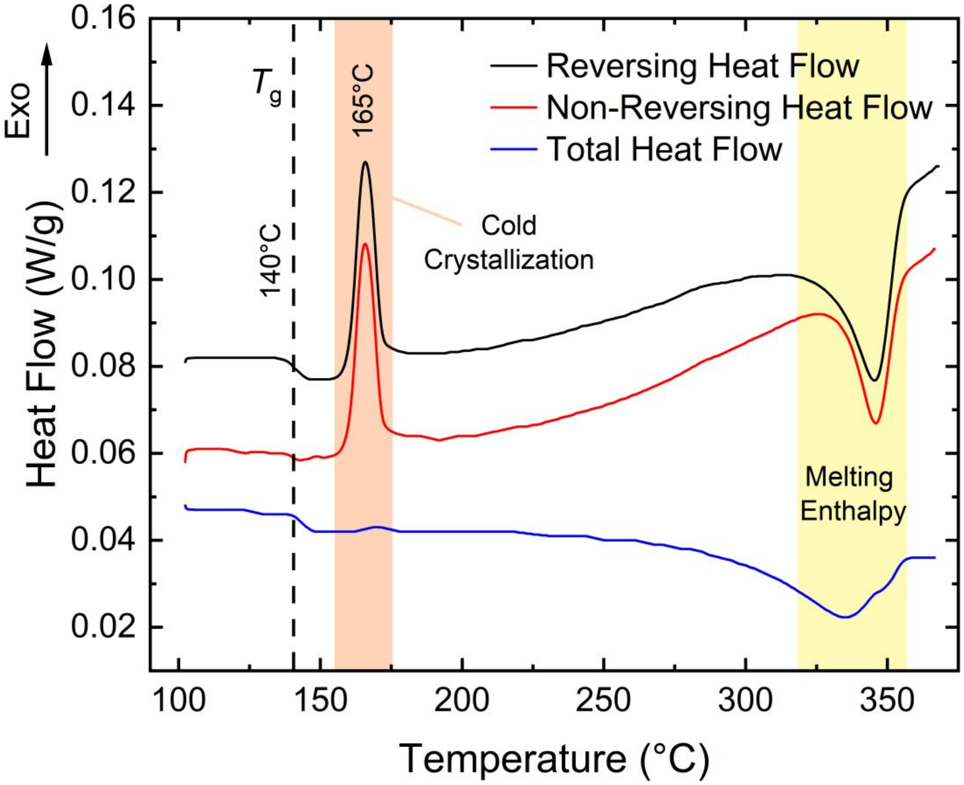

Figure S12. Modulated DSC heating scan of amorphous Victrex 450G PEEK films at 1 ˚C/min, amplitude of 0.21 ˚C, and period of 80 s, highlighting the differences in temperature of the glass transition temperature (T_g), cold crystallization peak, and melting enthalpy.

REFERENCES:

(1) Carrola, M.; Fallahi, H.; Koerner, H.; Pérez, L. M.; Asadi, A. Fundamentals of Crystalline Evolution and Properties of Carbon Nanotube-Reinforced Polyether Ether Ketone Nanocomposites in Fused Filament Fabrication. *ACS Applied Materials & Interfaces* **2023**, *15* (18), 22506-22523. DOI: 10.1021/acsami.3c01307.

(2) Foreman. J; Sauerbrunn, S. R. M., C. L. *Exploring the Sensitivity of Thermal Analysis Techniques to the Glass Transition*; TA Instruments, https://www.tainstruments.com/pdf/literature/TA082.pdf.

(3) Strasser, C. H., Jan. *Influence of the Cooling Rate on the Thermal Behavior of PET*; https://analyzing-testing.netzsch.com/en-US/application-literature/influence-of-the-cooling-rate-on-the-thermal-behavior-of-pet.
